# Distinct Roles of Type I and Type III Interferons in Intestinal Immunity to Homologous and Heterologous Rotavirus Infections

**DOI:** 10.1371/journal.ppat.1005600

**Published:** 2016-04-29

**Authors:** Jian-Da Lin, Ningguo Feng, Adrish Sen, Murugabaskar Balan, Hsiang-Chi Tseng, Constance McElrath, Sergey V. Smirnov, Jianya Peng, Linda L. Yasukawa, Russell K. Durbin, Joan E. Durbin, Harry B. Greenberg, Sergei V. Kotenko

**Affiliations:** 1 Department of Microbiology, Biochemistry and Molecular Genetics, New Jersey Medical School, Rutgers Biomedical and Health Sciences, Rutgers, Newark, New Jersey, United States of America; 2 Department of Pathology and Laboratory Medicine, New Jersey Medical School, Rutgers Biomedical and Health Sciences, Rutgers, Newark, New Jersey, United States of America; 3 Stanford University, Stanford, California, United States of America; 4 VA Palo Alto Health Care System, Palo Alto, California, United States of America; 5 Center for Immunity and Inflammation, New Jersey Medical School, Rutgers Biomedical and Health Sciences, Rutgers, Newark, New Jersey, United States of America; 6 University Hospital Cancer Center, New Jersey Medical School, Rutgers Biomedical and Health Sciences, Rutgers, Newark, New Jersey, United States of America; University of Freiburg, GERMANY

## Abstract

Type I (IFN-α/β) and type III (IFN-λ) interferons (IFNs) exert shared antiviral activities through distinct receptors. However, their relative importance for antiviral protection of different organ systems against specific viruses remains to be fully explored. We used mouse strains deficient in type-specific IFN signaling, STAT1 and Rag2 to dissect distinct and overlapping contributions of type I and type III IFNs to protection against homologous murine (EW-RV strain) and heterologous (non-murine) simian (RRV strain) rotavirus infections in suckling mice. Experiments demonstrated that murine EW-RV is insensitive to the action of both types of IFNs, and that timely viral clearance depends upon adaptive immune responses. In contrast, both type I and type III IFNs can control replication of the heterologous simian RRV in the gastrointestinal (GI) tract, and they cooperate to limit extra-intestinal simian RRV replication. Surprisingly, intestinal epithelial cells were sensitive to both IFN types in neonatal mice, although their responsiveness to type I, but not type III IFNs, diminished in adult mice, revealing an unexpected age-dependent change in specific contribution of type I versus type III IFNs to antiviral defenses in the GI tract. Transcriptional analysis revealed that intestinal antiviral responses to RV are triggered through either type of IFN receptor, and are greatly diminished when receptors for both IFN types are lacking. These results also demonstrate a murine host-specific resistance to IFN-mediated antiviral effects by murine EW-RV, but the retention of host efficacy through the cooperative action by type I and type III IFNs in restricting heterologous simian RRV growth and systemic replication in suckling mice. Collectively, our findings revealed a well-orchestrated spatial and temporal tuning of innate antiviral responses in the intestinal tract where two types of IFNs through distinct patterns of their expression and distinct but overlapping sets of target cells coordinately regulate antiviral defenses against heterologous or homologous rotaviruses with substantially different effectiveness.

## Author Summary

Two distinct families of interferons (IFNs), type I (IFN-α/β) and type III (IFN-λ) IFNs, are quickly produced in response to virus infection and engage distinct receptors to invoke shared rapid and broad-spectrum antiviral mechanisms against invading pathogens. However, the relative importance of type I and type III IFNs in protecting different organ systems against specific viruses or distinct strains of an individual virus remains to be fully explored. Here we demonstrated in suckling mice that neither type I nor type III IFNs are effective in blocking intestinal replication of murine rotavirus, rather, viral clearance is dependent upon adaptive immune responses. In contrast, both IFN types cooperate to control intestinal replication and extra-intestinal spread of simian rotavirus in neonatal mice. Unexpectedly, we found that although intestinal epithelial cells (IECs) respond to both types of IFNs in neonatal mice, responsiveness of IECs to type I IFNs, but not type III IFNs, is diminished in adult mice. Transcriptional analysis showed that both types of IFN receptors induced overlapping intestinal antiviral responses, which were abrogated only when both receptor types were deleted. Overall, these findings reveal a well-coordinated spatial and temporal regulation of antiviral defenses by type I and type III IFNs in the gastrointestinal tract that varies significantly depending on the viral strain examined.

## Introduction

Mucosal surfaces of mammalian reproductive, respiratory and gastrointestinal (GI) tracts are functionally unique. Most pathogens enter the host through mucosal surfaces, and epithelial cells lining these tracts serve as a first line of defense against invading pathogens. Moreover, mucosal surfaces are constantly exposed to a variety of microbes and therefore have the unique task of distinguishing between harmful pathogens and commensal symbiotic microbes. This challenge is particularly important in the GI tract where tolerance to billions of commensal microbes must be established and maintained. At the same time, the GI tract provides protection against pathogenic bacteria and GI viruses such as rotaviruses (RVs). RV infection causes severe diarrhea in infants and young children and is a major cause of morbidity and mortality in the developing world. The overall incidence of RV infection and morbidity appears to be similar in all unvaccinated areas, however the majority of RV-related deaths occur in developing countries [1, 2]. Although RV replicates primarily in the mature intestinal epithelial cells (IECs) of the small bowel, it can breach intestinal barriers and spread to the circulation and extra-intestinal organs (e.g. mesenteric lymph node (MLN), central nervous system (CNS), liver and biliary tree) [1, 3–5].

Initial antiviral protection in mammalian hosts is mainly dependent on the coordinated action of type I and type III IFNs, which are quickly produced by virus-infected and bystander IECs, as well as by intestinal hematopoietic cells [6–8]. These IFNs invoke innate antiviral mechanisms within virus-infected and uninfected bystander tissues, and coordinately regulate the development of adaptive immune responses against viral pathogens including RV [9–11]. Both IFN types activate the same signal transduction pathway which culminates in the formation of a ternary transcription complex, composed of STAT1, STAT2 and IRF9, and designated as IFN-Stimulated Gene Factor 3 (ISGF3) [12–14]. Subsequently, type I and type III IFNs induce expression of the same sets of IFN-stimulated genes (ISGs) and have very similar biological activities in sensitive cells [13, 15–17]. However, type I and type III IFNs engage distinct receptor complexes for their signaling. Whereas all type I IFNs utilize a heterodimeric receptor complex composed of IFN-αR1 and IFN-αR2 subunits, type III IFNs, or IFN-λs, engage the IFN-λR1 and IL-10R2 receptor chains for signaling [8, 12, 14, 18]. A major difference between the type I and type III IFN-based antiviral systems resides in the distinct cell-type specific pattern of receptor expression. In contrast to the type I IFN receptor that is ubiquitously expressed, the IFN-λ receptor is expressed primarily by epithelial cells [19].

Recent studies identified type III IFNs as critical non-redundant antiviral mediators in the GI tract. Type I IFNs alone were unable to restrict reovirus replication in the IECs of mice deficient in the type III IFN receptor [20]. Efficient control of murine norovirus, which replicates in the IECs, dendritic cells and B cells of the mouse, also required a functional IFN-λ receptor [21]. Furthermore, type III IFNs have recently been identified as unique antiviral mediators that were indispensable for the protection of suckling mice against infection with murine RV strain EDIM [22, 23]. However, the latter result contradicted other studies demonstrating that the murine EW-RV strain (derived from the original EDIM strain) replicated to a similar extent in wild-type (WT) or STAT1-deficient suckling mice due to its ability to effectively antagonize IFN production and signaling [6, 24–27]. In contrast, heterologous simian RV (RRV strain) was found to replicate poorly in WT mice, but RRV infection of STAT1-deficient suckling mice resulted in substantially enhanced intestinal replication and efficient systemic virus replication and disease [25, 26]. It was also demonstrated that despite their contrasting IFN-sensitive replication phenotypes, infection of suckling mice with either EW-RV or RRV results in similar induction of several IFN-stimulated genes (ISGs) in the small intestine at 16 hours post infection (hpi), confirming prior observations and indicating that RRV replication is uniquely sensitive to one or more of these antiviral effectors [6]. In fact, the substantial restriction of non-homologous RV strain replication in heterologous host species likely underlies the attenuating principle of several live RV vaccines that were based upon restricted replication of bovine, lamb or simian RV in humans [28, 29]. To further investigate the relative importance of type I and type III IFNs in regulating antiviral defenses in the GI tract, we utilized two distinct strains of RV, the homologous murine EW-RV strain and the heterologous simian RRV strain, and mice deficient in STAT1, type I or type III IFN receptors, or both types of IFN receptors.

Experiments reveal that neither type I nor type III IFNs alone, or both IFN types together were able to efficiently suppress the intestinal replication or diarrheal disease of murine EW-RV, demonstrating that homologous RV have evolved highly effective measures to circumvent the innate responses of their murine host. In contrast, we now demonstrate that both type I and type III IFNs are important mediators of antiviral protection of the GI tract and work cooperatively to limit intestinal replication of the heterologous simian RRV in suckling mice. Transcriptional analysis in the suckling mouse of bulk intestinal tissues revealed that similar patterns of ISG induction occurred in RRV-infected WT mice and in mice lacking either type I or type III IFN receptors, and induction of most ISGs was completely abolished in mice deficient in receptors for both IFN types. Further specific analysis demonstrated that IECs of neonatal mice were responsive to both types of IFNs as determined by immunohistochemical (IHC) staining for IFN-induced tyrosine phosphorylation and nuclear translocation of STAT1. In addition, pretreatment of neonatal mice with either type of IFN resulted in suppressed intestinal RRV replication. We also observed that responsiveness of IECs of adult mice to type I IFNs was diminished, whereas lamina propria cells (LPCs) of both neonatal and adult mice were responsive to type I but not type III IFNs. Both type I and type III IFNs helped to limit extra-intestinal RRV spread, but only type I IFNs were essential for controlling RRV replication in MLN. Our results reveal a previously underappreciated contribution of type I IFNs to the protection of IECs against GI viruses in neonatal mice, and demonstrate that both type I and type III IFNs act as important mediators of antiviral defenses within the GI tract, acting cooperatively to suppress heterologous RV replication in IECs and restrict extra-intestinal spread.

## Results

### Generation and characterization of IFN-λ receptor knockout (KO) mice

**Fig 1:**
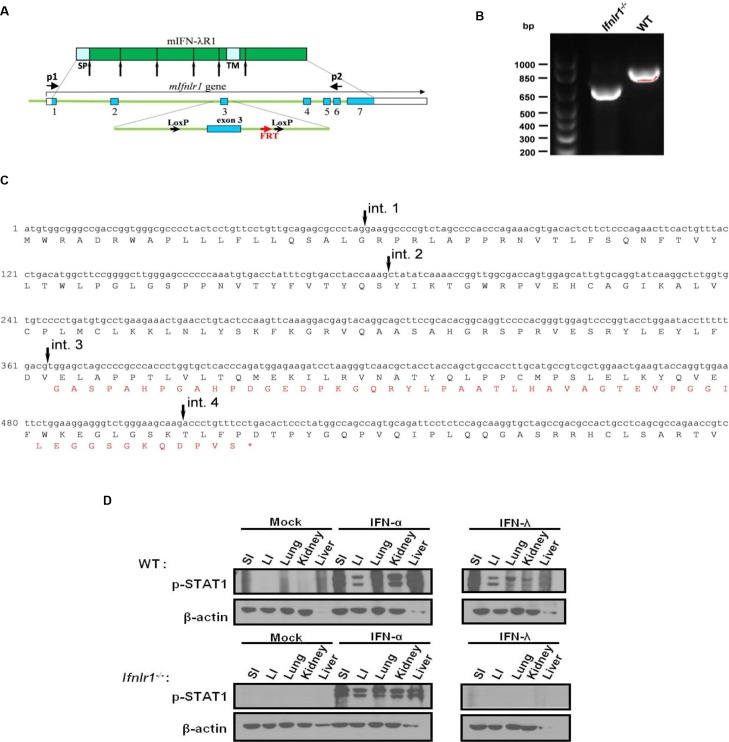
Generation and evaluation of IFN-λR1 KO mice. (A) The exon-intron structure of the *Ifnlr1* gene and the outline of the KO targeting vector are schematically shown. Exon 3 of the mouse *Ifnlr1* gene was flanked by two *loxp* sites introduced into corresponding introns away from the splice signals. Remaining FRT site and positions of two primers (p1 and p2) are schematically mapped. (B) RNA samples were obtained from kidney cells of WT and *Ifnlr1* ^*-/-*^ mice and used for RT-PCR with *Ifnlr1*-specific primers (p1 and p2) flanking exon 3. The sizes of the PCR fragments were determined by the agarose gel electrophoresis with 100 base pair DNA ladder shown in the left lane. (C) Sequencing of the PCR fragment from the *Ifnlr1* ^*-/-*^ mice revealed splicing of exon 2 into exon 4 of the *Ifnlr1* gene. A part of the *Ifnlr1* gene transcript with original reading frame (black letters) and a shifted reading frame with a premature stop codon (orange letters) that was generated as a result of exon 3 skipping are shown. Arrows indicated positions of introns 1 through 4. (D) 8-day-old WT and *Ifnlr1* ^*-/-*^ pups were intradermally injected with 1μg human IFN-αA/D (IFN-α) or murine IFN-λ2 (IFN-λ), 30 min later the indicated organs (small intestine (SI), large intestine (LI), lung, kidney and liver) were collected and analyzed for the presence of phosphorylated STAT1 (pSTAT1), as evidence of signaling through the IFN-αR or IFN-λR, by immunoblotting with pSTAT1-specific antibody. Immonoblotting with β actin antibody was used to evaluate equal loading.

To develop mice deficient in type III IFN signaling, exon 3 of the *IFNLR1* gene was targeted for elimination (Fig 1 and S1 Fig). LoxP sites flanking exon 3 were introduced into corresponding introns away from the splice signals to ensure that normal splicing of the modified *IFNLR1* gene is not disturbed (Fig 1A). The entire gene encoding IFN-λR1 consists of 7 exons. The deletion of exon 3 by Cre recombinase resulted in the generation of an abnormal IFN-λR1 transcript with exon 2 spliced to exon 4 leading to a reading frame shift and the premature termination of translation of the modified *IFNLR1* transcript (Fig 1B and 1C). Mice with the deleted exon 3 in the IFNLR1 gene had intact type I IFN signaling, but were unresponsive to type III IFNs as demonstrated by the inability of IFN-λ to trigger STAT1 phosphorylation in various tissues (Fig 1D). In addition, freshly isolated kidney cells from these mice up-regulated MHC class I antigen expression only in response to type I IFN, whereas cells from WT mice responded to both types of IFNs (S1C Fig).

### The homologous murine EW-RV strain effectively antagonizes IFN responses in suckling mice

Previous studies have shown that RV strains differ in their ability to antagonize IFN responses, both *in vitro* and *in vivo*, in part dependent on the species origin of the virus and the host [6, 30–34]. RV strains are best able to circumvent innate immune responses in their natural, homologous species host. For example, although both heterologous simian RRV and homologous murine EW-RV induce similar levels of type I IFNs and several ISGs in the small intestine at 16 hpi, replication of RRV was highly sensitive to IFN-mediated antiviral defenses and occurred much more efficiently in the intestine of type I IFN receptor and STAT1 KO suckling mice than in WT mice [6, 25], whereas murine EW-RV strain was able to replicate comparably in the intestine of suckling mice in the presence or absence of IFNs; EW-RV shedding and clearance proceeded at similar rates in wild type and STAT1 KO mice, which are deficient in type I, II and III IFN signaling [6, 24, 26]. In contrast to these results with STAT1 and type I IFN receptor KO mice, two recent studies with IFN receptor-deficient animals indicated that type III IFNs, and not type I IFNs, could mediate very significant and biologically relevant innate antiviral protection of neonatal mice IECs during murine RV infection [22, 23]. In this studies, it was observed that the action of type III IFNs alone was sufficient to substantially restrict murine RV (EDIM-RV strain) intestinal replication and virus shedding while promoting suckling mouse weight gain and diminishing diarrheal disease. On the other hand, as previously reported by others [6, 24, 26], type I IFNs did not appear to play a substantial role in intestinal epithelial antiviral defenses, viral replication, or in protecting the suckling mice from murine RV associated disease [22, 23].

**Fig 2:**
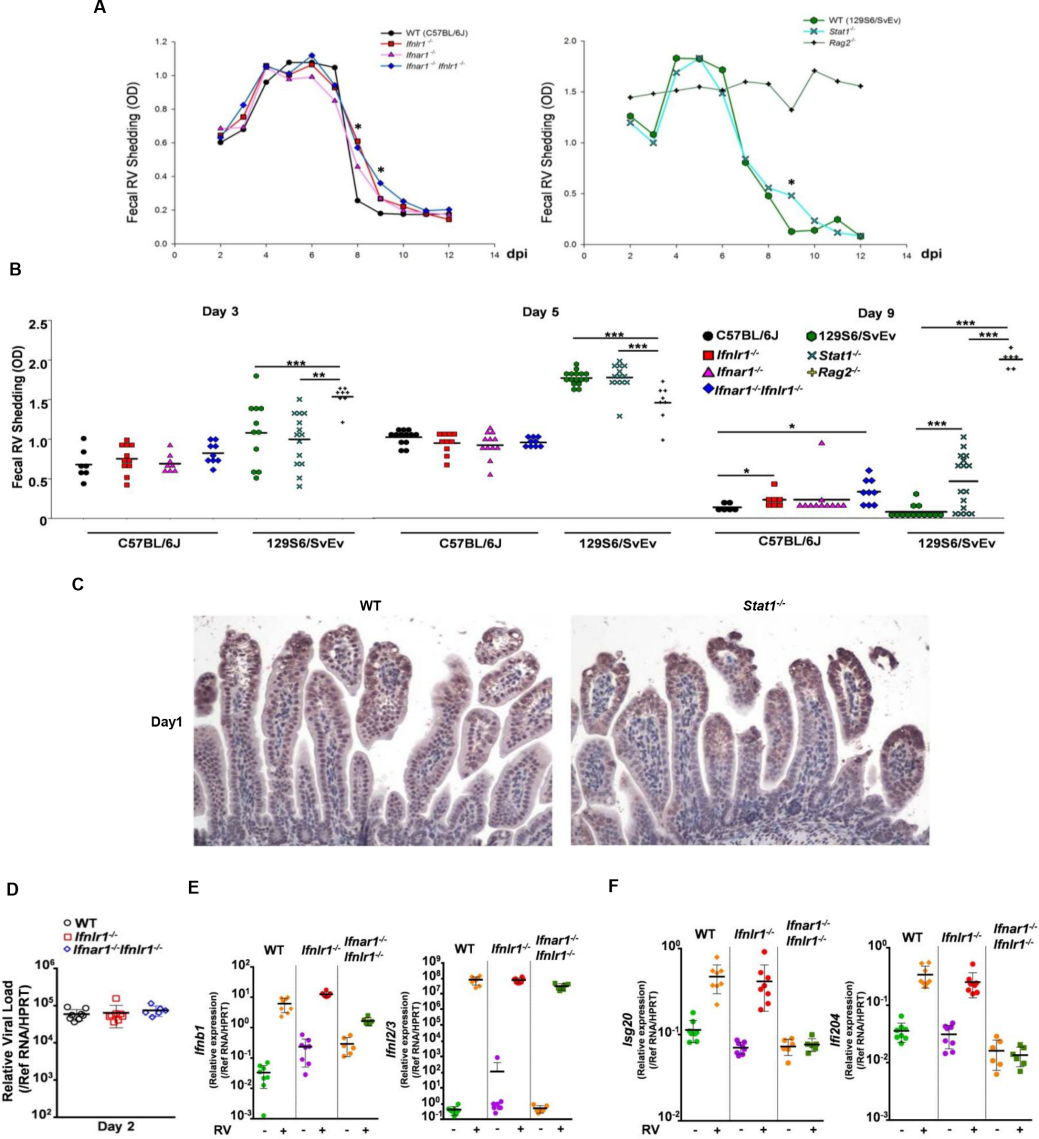
Murine EW-RV efficiently replicates in intestine of suckling mice, regardless of the IFN action. (A and B) Eight-day-old suckling WT (n = 6–13), *Ifnar1* ^*-/-*^ (n = 7–11), *Ifnlr1* ^*-/-*^ (n = 8–12), *Ifnar1* ^*-/-*^ *Ifnlr1* ^*-/-*^ (n = 8–12) mice (on C57BL/6J background) and WT (n = 11–16), *Stat1* ^*-/-*^ (n = 13–16), *Rag2* ^*-/-*^ (n = 6–8) mice (on 129S6/SvEv background) were orally infected with 10^4^ DD_50_ EW-RV. Stool samples were collected daily from 2 to 12 dpi, and EW-RV fecal shedding determined by ELISA and expressed as OD unit. The variable sample number (n) reflects the variation of animals per time point. (A) Graph of kinetics of mean fecal EW-RV shedding in indicated strains of mice on C57BL/6J or 129S6/SvEv background. (B) Dot plot presentations of EW-RV shedding in indicated mouse strains on 3, 5 and 9 dpi. (C) Representative immunohistochemical staining of EW-RV in small intestine of WT and *Stat1* ^*-/-*^ mice (on 129S6/SvEv background) on 1 dpi. (D-F) Graph of (D) quantitative RT-PCR detection of EW-RV levels, (E) IFN expression and (F) expression of indicated ISGs in small intestine of EW-RV-infected mice on 2 dpi. Each symbol (B and D-F) represents an individual mouse; horizontal lines indicate the mean (± SEM). *: significant difference (*P* < 0.05); **: significant difference (*P* < 0.01); ***: significant difference (*P* < 0.001).

In order to better clarify the conflicting data in the reports that found no substantial changes on murine RV replication or disease in suckling *Stat1* ^*-/-*^ mice and the reports that found that murine RV replication and disease was substantially augmented in *Ifnlr1* ^*-/-*^ mice, eight-day-old WT, *Ifnlr1* ^*-/-*^, *Ifnar1* ^*-/*^ and *Ifnar1* ^*-/-*^ *Ifnlr1* ^*-/-*^ mice (on the C57BL/6J background) and WT, *Stat1* ^*-/-*^ and *Rag2* ^*-/-*^ mice (on 129S6/SvEv background) were orally inoculated with 10^4^ diarrhea dose 50 (DD_50_) of the murine EW-RV strain derived from the original EDIM-RV isolate [33], and fecal EW-RV shedding was initially quantified by ELISA. We observed virtually identical fecal shedding of EW-RV in WT, *Ifnlr1* ^*-/-*^, *Ifnar1* ^*-/-*^, *Ifnar1* ^*-/-*^ *Ifnlr1* ^*-/-*^ or *Stat1* ^*-/-*^ mice during the first 7 days post infection (dpi) (Fig 2A and 2B). Slightly delayed viral clearance in suckling mice deficient in type I, type III, or both IFN receptors, together with small differences in virus shedding, was observed on 8 and 9 dpi (Fig 2A and 2B). In agreement with our previous studies [24–26], we saw little difference in the kinetics of EW-RV clearance between 129S6/SvEv WT and *Stat1* ^*-/-*^ suckling mice, with a slight increase in shedding only detected in *Stat1* ^*-/-*^ mice on 9 dpi (Fig 2A and 2B). Consistent with this observation, similar levels of EW-RV protein were detected in the small intestine of infected WT and *Stat1* ^*-/-*^ mice on 1 dpi (Fig 2C). Of note, unresolved shedding was observed only in *Rag2* ^*-/-*^ animals (Fig 2A and 2B), demonstrating that adaptive, rather than innate, immune responses are primarily responsible for resolving EW-RV infection.

We also detected similar patterns of viral replication in small intestines of EW-RV-infected C57BL/6J WT, *Ifnlr1* ^*-/-*^ and *Ifnar1* ^*-/-*^ *Ifnlr1* ^*-/-*^ mice, as measured by qRT-PCR (Fig 2D). Although IFN-λ transcripts were strongly up-regulated to similar levels in intestines of all mouse strains examined, IFN-β transcripts were induced less efficiently with considerably weaker IFN-β induction in *Ifnar1* ^*-/-*^ *Ifnlr1* ^*-/-*^ mice than in WT or *Ifnlr1* ^*-/-*^ mice (Fig 2E), possibly due to the absence of a positive feedback loop in these animals. ISG induction occurred with similar efficiency in WT and *Ifnlr1* ^*-/-*^ mice, but was completely abrogated when both IFN-λ and IFN-α receptors were lacking (Fig 2F). Thus, intestinal ISG expression following homologous RV infection can occur in the absence of IFN-λ receptor-mediated signaling. Nevertheless, the presence of IFN and ISG expression has minimal effect on EW-RV replication in small intestine of IFN receptor-sufficient or deficient mice (Fig 2A–2D and 2F), demonstrating that EW-RV efficiently antagonizes most IFN-mediated antiviral responses [27]. These data obtained in both *Ifnlr1* ^*-/-*^ and *Ifnar1* ^*-/-*^ *Ifnlr1* ^*-/-*^ mice and confirmed in *Stat1* ^*-/-*^ mice differ from the results of recently published studies, where suckling *Ifnlr1* ^*-/-*^ and *Ifnar1* ^*-/-*^ *Ifnlr1* ^*-/-*^ mice were found to be substantially more susceptible to the murine EDIM-RV strain, and type III IFNs were postulated to be the primary mediators of ISG expression in the intestinal epithelium [22, 23]. In these conflicting studies, mice reconstituted with a functional *Mx1* gene were used. In our studies, all the mouse strains examined were on either C57BL/6J or 129S6/SvEv backgrounds and lacked the functional *Mx1* gene. However, conventional C57BL/6J mice that are deficient in *Mx1*, and *Mx1*-reconstituted C57BL/6J mice showed no differences in either EW-RV replication or in their patterns of IFN and ISG induction (S2A–S2D Fig), ruling out the possibility that Mx1 was responsible for the observed differences between these studies. We also obtained the murine EDIM-RV strain that was used in the conflicting studies [22, 23] and compared it to our murine EW-RV strain that was also derived from the original EDIM strain. Both strains replicated similarly in WT or *Stat1* ^*-/-*^ 129S6/SvEv or C57BL/6J WT suckling mice (S3A and S3B Fig), indicating that differences in the replication phenotype of murine RV in the two studies were not likely due to viral strain variations. Overall, EW-RV replication in suckling mice was not significantly affected by the presence of either type I or type III IFNs, confirming the substantial insensitivity of murine RV to IFN-mediated antiviral effects on virus replication in the suckling murine host [6, 24, 26].

### IFNs affect diarrheal disease but not weight gain during RV infection

**Fig 3:**
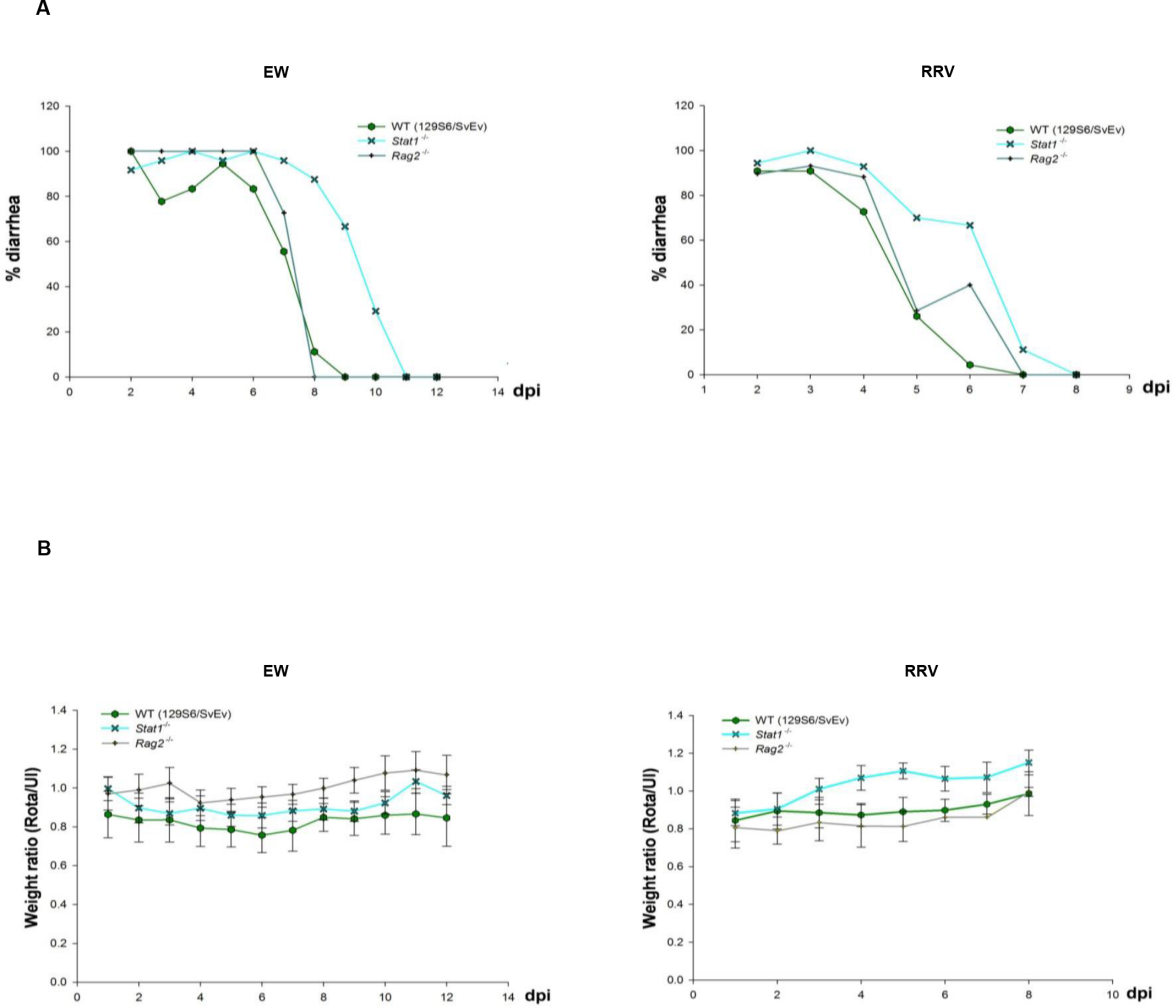
IFNs may affect diarrheal disease but not weight gain during RV infection. Eight-day-old suckling 129S6/SvEv, *Stat1* ^*-/-*^ and *Rag2* ^*-/-*^ (on 129S6/SvEv background) mice (n = 6–10) were orally infected with 10^4^ DD_50_ EW-RV or 4x10^6^ FFU RRV. The variable sample number (n) reflects the variation of animals per time point. (A) Graph of average diarrheal disease and (B) average ratio of body weight between RV-infected and uninfected suckling mice were monitored daily in EW-RV (2 to 12 dpi) or RRV-infected suckling mice (2 to 8 dpi).

A previous study also revealed substantial growth retardation of EDIM-RV-infected *Ifnlr1* ^*-/-*^ pups compared to their WT counterparts, and correlated these differences with increased EDIM-RV replication in *Ifnlr1* ^*-/-*^ mice [23]. To determine whether lack of IFN signaling might affect pathophysiologic parameters other than RV replication, weight gain and diarrheal disease were also monitored in EW-RV-infected suckling WT, *Stat1* ^*-/-*^ and *Rag2* ^*-/-*^ mice (on 129S6/SvEv background). Diarrhea appeared on 2 dpi in all groups, affected virtually all inoculated pups, and resolved between 8 and 11 dpi (Fig 3A), with no difference in the numbers of animals affected, despite continuous virus shedding by *Rag2* ^*-/-*^ mice (Figs 2A and 3A). However, diarrhea was moderately prolonged in the EW-RV-infected *Stat1* ^*-/-*^ mice (Fig 3A), suggesting that IFN signaling may affect the duration of murine RV-associated diarrheal disease. Furthermore, similarly delayed resolution of diarrhea was observed in *Stat1* ^*-/-*^ mice infected with simian RRV (Fig 3A). Despite moderately prolonged diarrhea in *Stat1* ^*-/-*^ animals or the continued EW-RV shedding in *Rag2* ^*-/-*^ mice, body weight gain of WT, *Stat1* ^*-/-*^ and *Rag2* ^*-/-*^ mice in either EW-RV or RRV-infected groups remained similar (Fig 3B). These experiments suggest that RV-induced diarrhea and weight gain are not necessarily correlated with virus load, since the chronically infected *Rag2* ^*-/-*^ mice (Fig 2A) resolved diarrhea earlier than EW-RV or RRV-infected *Stat1* ^*-/-*^ pups and exhibited body weight gain comparable to WT mice (Fig 3A). Therefore, although IFNs may be involved in the timely resolution of murine RV-induced diarrhea, this is not directly correlated with either virus load or weight gain. Of interest, although the level of shedding and the severity of diarrheal disease have been directly correlated in children [35], this correlation is not invariable since the *Rag2* ^*-/-*^ mice resolved diarrhea while continuing to shed RV (Figs 2 and 3).

### Both type I and type III IFNs contribute to the restriction of simian RRV replication in the intestine

**Fig 4:**
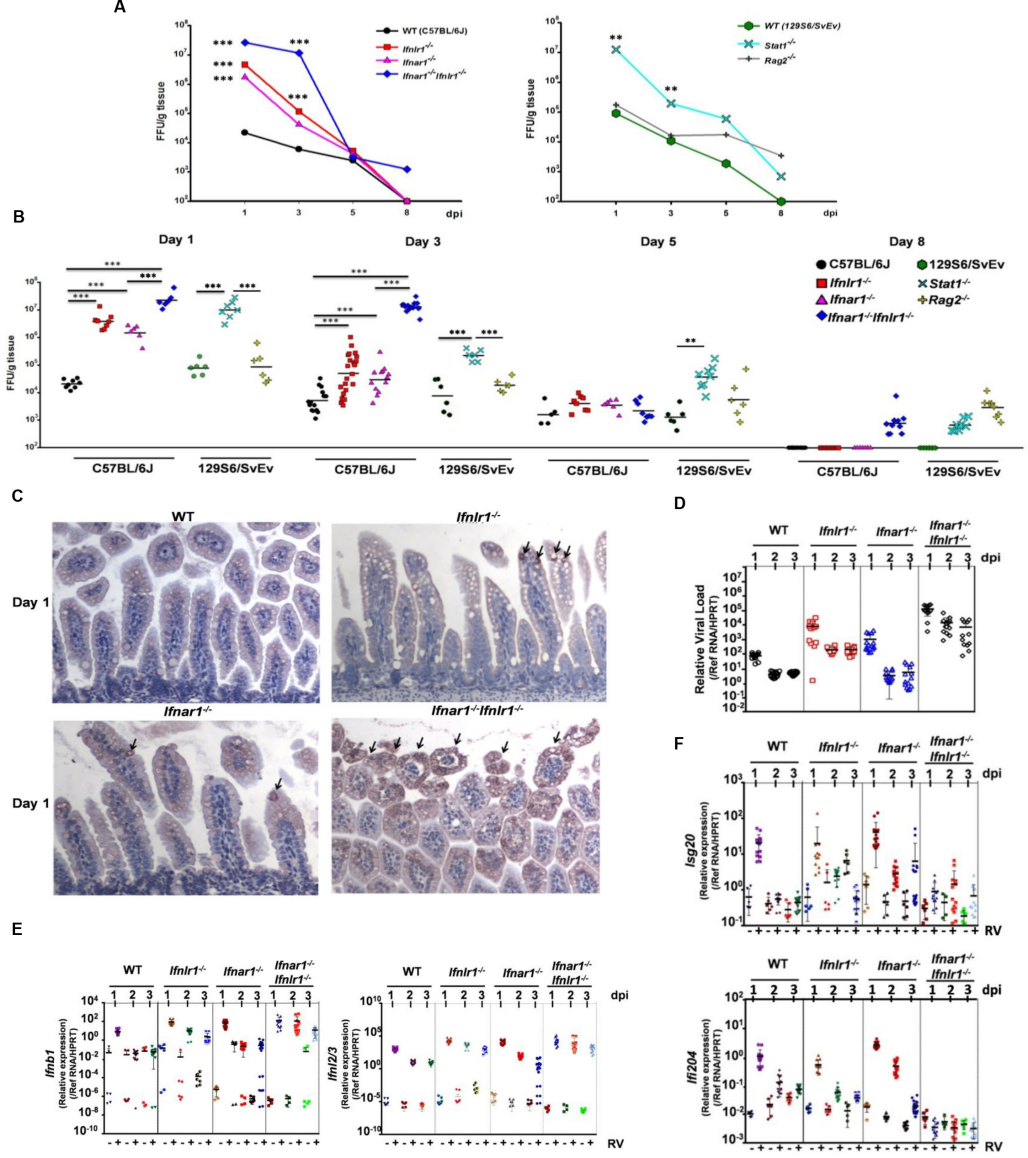
Both type I and type III IFNs contribute to intestinal antiviral immunity of suckling mice to simian RRV. Eight-day-old suckling WT (n = 5–14), *Ifnar1* ^*-/-*^ (n = 6–12), *Ifnlr1* ^*-/-*^ (n = 8–20) and *Ifnar1* ^*-/-*^ *Ifnlr1* ^*-/-*^ mice (n = 6–10) (on C57BL/6J background), and WT (n = 6), *Stat1* ^*-/-*^ (n = 6–8) and *Rag2* ^*-/-*^ mice (n = 6) (on 129S6/SvEv background) were orally infected with 4x10^6^ FFU RRV. Small intestines were collected at indicated dpi and virus titers were determined by immunohistochemical infectious focus assay and expressed as FFU/g of tissue. The variable sample number (n) reflects the variation of animals per time point. (A) Graph of mean kinetics of RRV replication in small intestine of suckling mice of various strains on C57BL/6J or 129S6/SvEv background. (B) Dot plot presentations of intestinal RRV titers in suckling mice of indicated strains on 1, 3, 5 and 8 dpi. (C) Representative immunohistochemical staining of RRV antigens in small intestine of RRV-infected C57BL/6J WT, *Ifnlr1* ^*-/-*^, *Ifnar1* ^*-/-*^ and *Ifnar1* ^*-/-*^ *Ifnlr1* ^*-/-*^ mice on 1 dpi. (D-F) Quantitative RT-PCR detection of (D) RRV levels, (E) IFN expression and (F) expression of indicated ISGs in small intestine of RRV-infected mice on 1, 2 and 3 dpi. Each symbol (B and D-F) represents an individual mouse; horizontal lines indicate the mean (± SEM). *: significant difference (*P* < 0.05); **: significant difference (*P* < 0.01); ***: significant difference (*P* < 0.001).

Because homologous murine RV is remarkably resistant to IFN-mediated innate responses in suckling mice (Fig 2) [6, 21–23] and because prior studies had indicated that heterologous RVs might be more responsive to IFN mediated suppression [5, 6, 25], we next examined the heterologous simian RRV to assess the relative contributions of type I and type III IFNs to the control of non-murine RV replication and clearance. Eight-day-old suckling WT mice or mice deficient in IFN type-specific signaling (*Ifnlr1* ^*-/-*^, *Ifnar1* ^*-/-*^ and *Ifnar1* ^*-/-*^ *Ifnlr1* ^*-/-*^ mice, all on C57BL/6J background) were orally inoculated with 4 x 10^6^ FFU RRV. Intestinal samples were collected and RV titers determined on 1, 3, 5 and 8 dpi. On 1 and 3 dpi, there were no significant differences in intestinal virus replication between *Ifnlr1* ^*-/-*^ and *Ifnar1* ^*-/-*^ mice, whereas either type I or type III IFN receptor-deficient (*Ifnar1* ^*-/-*^ or *Ifnlr1* ^*-/-*^) animals supported significantly greater intestinal RRV replication (>100 fold) than did WT mice (Fig 4A and 4B). Importantly, RRV replicated to significantly higher titers in *Ifnar1* ^*-/-*^ *Ifnlr1* ^*-/-*^ and *Stat1* ^*-/-*^ mice than in mice lacking either IFN receptor alone (Fig 4A and 4B). *Ifnar1* ^*-/-*^ *Ifnlr1* ^*-/-*^ and *Stat1* ^*-/-*^ mice also showed delayed virus clearance, with virus still present in the small intestine on 8 dpi, a time point when virus could no longer be detected in WT, *Ifnar1* ^*-/-*^ and *Ifnlr1* ^*-/-*^ strains (Fig 4A and 4B). Low, but sustained, RRV levels were detected in the small intestine of *Rag2* ^*-/-*^ mice from 1 to 8 dpi (Fig 4A and 4B), which persisted through 15 dpi. Consistent with the virus titer results, infected IECs were rarely detected in the small intestines of RRV-infected *Ifnar1* ^*-/-*^ and *Ifnlr1* ^*-/-*^ mice by immunohistochemistry, with much more extensive antigen-staining present in the IECs of infected *Ifnar1* ^*-/-*^ *Ifnlr1* ^*-/-*^ animals (Fig 4C). Viral antigen was found primarily in IECs at the tips of the villi in type I or type III IFN receptor-deficient mice, and RRV-infected IECs were essentially absent in infected WT mice. These results indicate that both type I and type III IFNs independently restrict replication of the heterologous simian RRV in intestines of suckling mice, with resolution of infection mediated primarily by the adaptive immune response.

RV gene transcriptional analysis revealed robust RRV replication in *Ifnar1* ^*-/-*^ *Ifnlr1* ^*-/-*^ mice, with incremental decreases in replication occurring in the single IFN receptor KO and WT pups, respectively (Fig 4D). The induction of IFN-β transcripts by RRV in WT mice occurred primarily on 1 dpi. In comparison, in mice lacking receptors for either type I or type III IFNs, as well as in the double IFN receptor KO mice, IFN-β induction was more robust and occurred over a prolonged period of time following RRV infection, particularly in *Ifnar1* ^*-/-*^ *Ifnlr1* ^*-/-*^ mice and to a lesser extent in *Ifnlr1* ^*-/-*^ mice (Fig 4E). Similar expression patterns were observed for IFN-λ transcripts but with sustained up-regulation of IFN-λ expression on 2 and 3 dpi in all mouse strains (Fig 4E). Similar to IFN-β, expression levels of IFN-λ transcripts were elevated in all KO strains in comparison to WT mice, and sustained elevated expression of IFN-λ transcripts was mostly pronounced in either *Ifnar1* ^*-/-*^ *Ifnlr1* ^*-/-*^ or *Ifnlr1* ^*-/-*^ mice. Patterns of IFN expression (Fig 4E) mirrored the transcriptional RRV load (Fig 4D), suggesting that increased viral replication in the absence of the cognate IFN receptors and their effector pathways triggers prolonged and elevated expression of both IFN types in IFN receptor deficient mice. Because expression of IFN transcripts was similar in response to RRV infection of WT and single or double IFN receptor-deficient mice (Fig 4E), type I and type III IFNs appear to be induced independently during RV infection. To assess whether much more robust up-regulation of *Ifnl* transcription compared to *Ifnb* transcription correlates with higher levels of type III IFN protein expression, homogenates of small intestines from RRV-infected mice were collected on 1 dpi and used for IFN-λ ELISA and type I IFN bioassay (S4 Fig **).** Whereas IFN-λ proteins were detected at about 300 to 500 pg per 100 mg of tissue, levels of type I IFNs were below the detection level of the bioassay (<30 units/ml; ~300 pg per 100 mg). These results correlate with our transcriptional analyses and indicate that RRV infection predominantly triggers production of type III IFNs in the small intestine. Both the magnitude and kinetics of ISG expression were similar in WT and single IFN receptor-deficient mice (Fig 4F), demonstrating that either type I or type III IFN can up-regulate ISG expression in small intestine of RRV-infected mice independently. Moreover, the increased expression of these ISGs was abolished in double IFN receptor KO mice after RRV infection, despite the induction of type I and type III IFN transcripts (Fig 4E and 4F). The diminished ISG induction in *Ifnar1* ^*-/-*^ *Ifnlr1* ^*-/-*^ mice correlated with increased viral replication in these animals, reflecting the sensitivity of RRV to IFN-mediated innate antiviral defenses. The delayed RRV clearance in *Ifnlr1* ^*-/-*^ mice (Fig 4D) correlated with only a modest induction of *Ifnb* (Fig 4E) and the lack of transcriptional *Ifna* responses (S5A–S5C Fig). In contrast, levels of *Ifnl2/3* transcription were substantially elevated in response to RRV infection (Fig 4E) and correlated with fast reduction of RRV by 2 dpi in *Ifnar1* ^*-/-*^ mice (Fig 4D), suggesting a predominant role of type III IFNs in the intestinal antiviral defense.

### Responsiveness of IECs to IFNs is age-dependent

**Fig 5:**
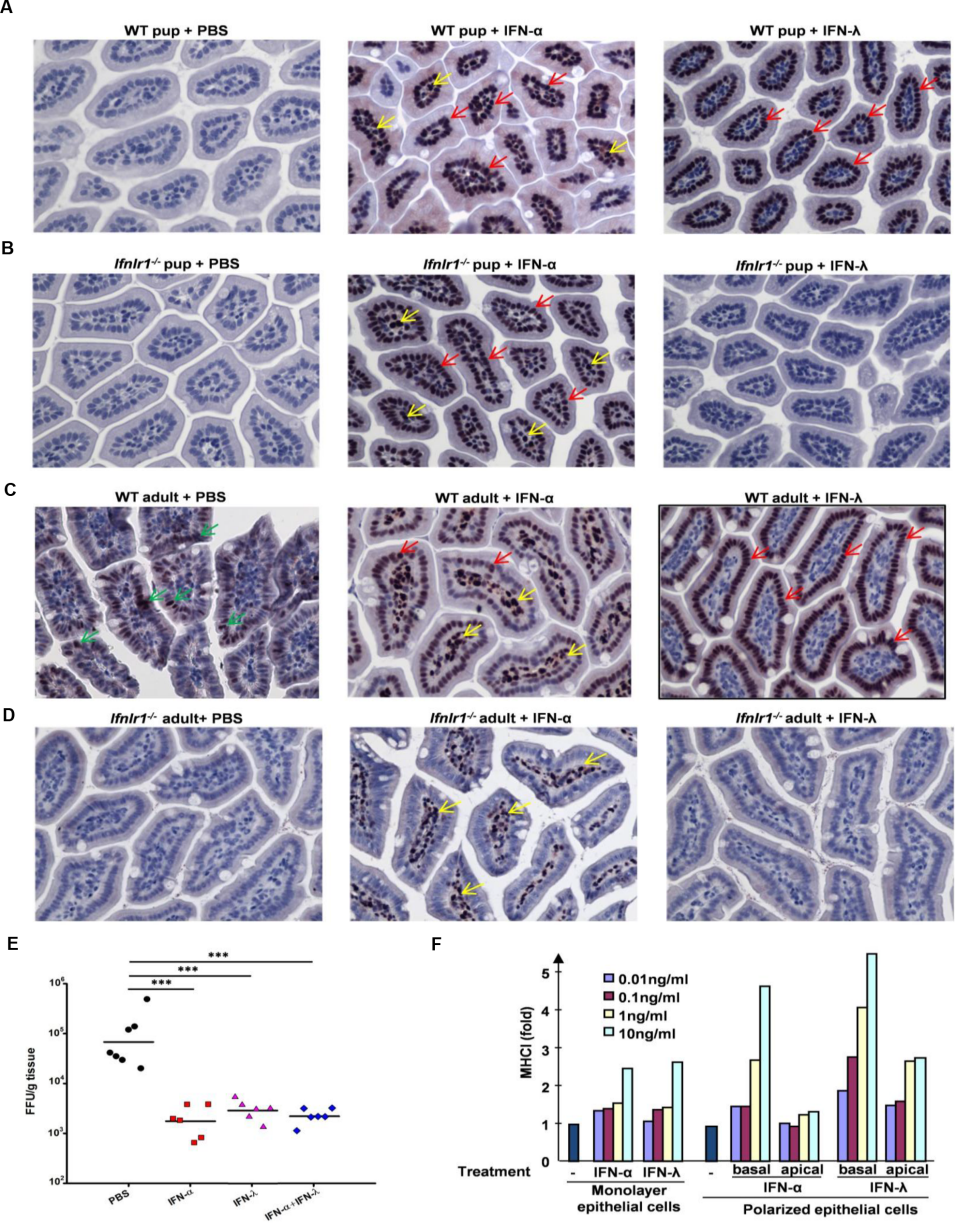
Differential responsiveness of IECs and LPCs to type I and type III IFNs. (A-C) Representative of immunohistochemical staining of pSTAT1 (pTyr701) in small intestine of eight-day-old suckling (A) C57BL/6J WT or (B) *Ifnlr1* ^*-/-*^ mice, or six to eight-week-old (C) C57BL/6J WT mice or (D) *Ifnlr1* ^*-/-*^ mice were subcutaneously injected with PBS, human IFN-αA/D (IFN-α; 1μg) or murine IFN-λ2 (IFN-λ; 1μg). Small intestine samples were collected 30 min post injection. Red and yellow arrows indicate nuclear staining of pSTAT1 in IFN-treated IECs and LPCs, respectively. Green arrows indicate low levels of pSTAT1 staining in PBS-treated IECs in adult WT mice. (E) Graph of virus titers on 1 dpi in small intestine of eight-day-old suckling C57BL/6J WT mice subcutaneously injected with PBS, human IFN-αA/D (IFN-α; 1μg) or murine IFN-λ2 (IFN-λ; 1μg), or their combination (1μg of each IFN) 6 h before oral infection with 4x10^6^ FFU RRV. Virus titers were determined by immunohistochemical focus assay and expressed as FFU/g of tissue. (F) Graph of fold increase of MHC class I expression in human SW-1116 cells grown under polarized or regular culture conditions, treated at the apical or basolateral surfaces with various amounts of human IFN-αA/D or IFN-λ1 as indicated for 72 h. One representative experiment out of two is shown.

Because RRV replication was significantly increased in *Ifnar1* ^*-/-*^ *Ifnlr1* ^*-/-*^ and *Stat1* ^*-/-*^ animals when compared to single IFN receptor KO mice, it seemed likely that IECs in suckling mice can respond to either IFN-α or IFN-λ. Such a possibility is also supported by the abrogation of RRV-mediated intestinal ISG expression only in the absence of receptors for both IFN types. Signaling downstream of either the type I or type III IFN receptor leads to tyrosine phosphorylation of STAT1 (pSTAT1). To directly investigate responsiveness of IECs and cells within the lamina propria to IFNs, eight-day-old suckling C57BL/6J mice were subcutaneously injected with PBS, IFN-α, or IFN-λ, and levels and nuclear translocation of pSTAT1 in small intestine were assessed by immunohistochemical staining with pSTAT1 specific antibody (Fig 5A). Both IECs and LPCs of the small intestine of suckling mice were responsive to IFN-α, whereas only IECs were responsive to IFN-λ (Fig 5A). To exclude the possibility that lack of type III IFN signaling might alter responsiveness of IECs to type I IFNs, *Ifnlr1* ^*-/-*^ suckling mice were also treated with IFN-α and STAT1 phosphorylation was again examined in the small intestine. Type I IFN-induced pSTAT1 was detected in both IEC and LPC compartments while no pSTAT1 staining was found in response to IFN-λ treatment (Fig 5B). Therefore, in the suckling mouse, both type I and type III IFNs are capable of triggering STAT1 activation in IECs, whereas LPCs are only responsive to type I IFNs.

These data from suckling mice are different from previous observations wherein mouse IECs were found to be unresponsive to type I IFNs when adult mice were treated with plasmid-delivered IFNs [20, 22]. To investigate whether the IFN responsiveness of IECs might be age-dependent, IFN-mediated STAT1 activation was subsequently assessed in six to eight-week-old WT or *Ifnlr1* ^*-/-*^ mice. Only LPCs, but not IECs, were strongly responsive to type I IFNs in the older mice, whereas responsiveness of IECs to type III IFNs remained robust in adult animals (Fig 5C and 5D). Low levels of STAT1 phosphorylation were detected in PBS-treated IECs in adult WT mice, but not in mice deficient in type III IFN receptor (Fig 5C and 5D), suggesting that weak, constitutive IFN-λ signaling is likely to be maintained in IECs in adult mice. These results reveal an unexpected age-related change in the type I IFN responsiveness of IECs, which is robust in early post-natal life, and strongly diminished as the mouse matures.

To directly assess whether STAT1 activation in IECs correlates with antiviral protection, eight-day-old suckling mice were subcutaneously injected with either IFN-α or IFN-λ 6 h before RRV infection, and virus replication was analyzed on 1 dpi (Fig 5E). Pretreatment with either type of IFN resulted in reduced RRV levels on 1 dpi, demonstrating that both type I and type III IFNs inhibit intestinal RRV replication. In a previous study, unresponsiveness of IECs to systemic type I IFN treatment was explained by the polarized nature of IFN signaling in IECs [22]. In that study, polarized IECs were shown to respond to type I IFNs only when IFN-β was delivered apically, whereas type III IFNs were active on both basolateral and apical surfaces [22]. In contrast, our experiments with human SW-1116 colorectal carcinoma cells demonstrated that upon polarization, these cells strongly respond to either type I or type III IFNs only basolaterally (Fig 5F). Of note, sensitivity of SW-1116 cells to IFN-λ was enhanced upon polarization to higher degree than that to IFN-α, and weak responsiveness to IFN-λ at the apical surface was also detected (Fig 5F). Overall, these results demonstrate that type I and type III IFNs are capable of inducing antiviral protection in IECs of suckling mice in a redundant manner.

### Type I IFNs play a major role in controlling extra-intestinal spread and replication of RRV in MLN

**Fig 6:**
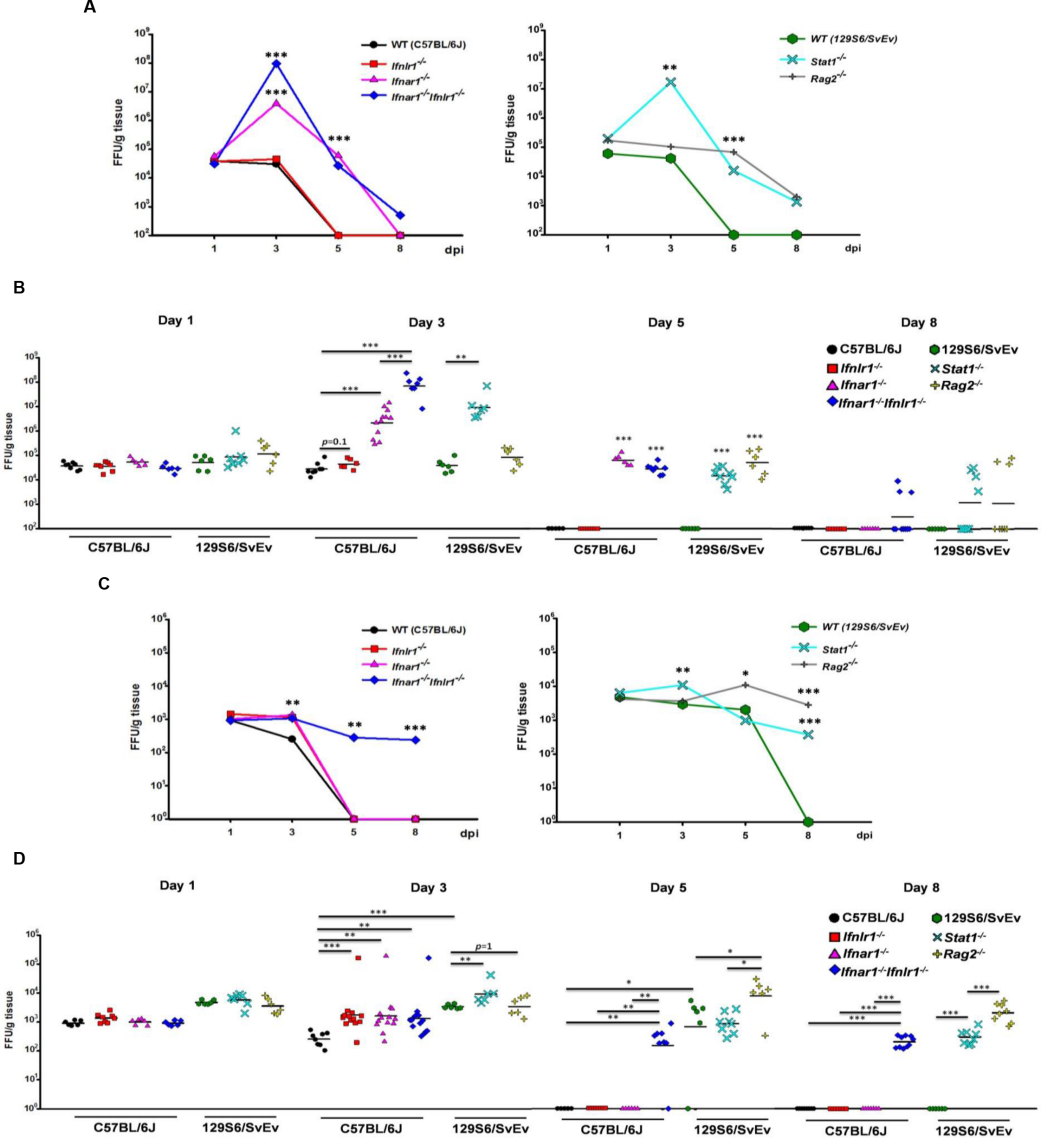
Type I IFNs control extra-intestinal spread and replication of simian RRV in MLN, but both IFN types limit simian RRV replication in liver. Eight-day-old suckling WT (n = 6–8), *Ifnar1* ^*-/-*^ (n = 6–13), *Ifnlr1* ^*-/-*^ (n = 6–13) and *Ifnar1* ^*-/-*^ *Ifnlr1* ^*-/-*^ (n = 6–13) mice (on C57BL/6J background), and WT (n = 6), *Stat1* ^*-/-*^ (n = 6–8) and *Rag2* ^*-/-*^ mice (n = 6–8) (on 129S6/SvEv background) were orally infected with 4x10^6^ FFU RRV. MLN and liver samples were collected at indicated dpi and virus titers were determined by immunohistochemical infectious focus assay and expressed as FFU/g of tissue. The variable sample number (n) reflects the variation of animals per time point. (A and C) Graph of mean kinetics of RRV replication in MLN (A) and liver (C) of suckling mice of indicated strains on C57BL/6J or 129S6/SvEv background. (B and D) Dot plot presentations of RRV titers in MLN (B) and liver (D) of suckling mice of indicated strains on 1, 3, 5 and 8 dpi. Each symbol (B and D) represents an individual mouse; horizontal lines indicate the mean (± SEM).*: significant difference (*P* < 0.05); **: significant difference (*P* < 0.01); ***: significant difference (*P* < 0.001).

Previous mouse studies demonstrated that RRV can spread to and replicate in extra-intestinal sites as efficiently as murine RV including MLN, and that type I IFNs are important for restricting RRV replication and pathogenesis at these sites [4, 5, 25]. To further investigate the role of specific IFNs in controlling early extra-intestinal spread and replication, RRV titers in MLN of infected mice were assayed. There were no significant differences between virus titers in MLN of RRV-infected WT and IFN receptor-deficient animals on 1 dpi (Fig 6A). However, by 3 dpi, elevated virus titers on the order of 100-fold greater than WT were detected in MLN of RRV-infected *Ifnar1* ^*-/-*^ mice, and 1,000-fold above WT levels in *Ifnar1* ^*-/-*^ *Ifnlr1* ^*-/-*^ animals (Fig 6B). RRV replication was still detectable in MLN of *Ifnar1* ^*-/-*^ and *Ifnar1* ^*-/-*^ *Ifnlr1* ^*-/-*^ mice on 5 dpi, and MLN from several *Ifnar1* ^*-/-*^ *Ifnlr1* ^*-/-*^ mice were still RV positive on 8 dpi (Fig 6A and 6B). Levels of RRV were not elevated above WT controls in the *Ifnlr1* ^*-/-*^ mice. Therefore, type I IFNs mediate the primary control of extra-intestinal spread and replication of RRV in MLN, although a further deficiency in type III IFN signaling enhances virus replication in MLNs when combined with a type I IFN deficiency at early times post infection. Consistent with these data, elevated virus titers were also found in MLN of RRV-infected *Stat1* ^*-/-*^ mice on 3 dpi, and virus was still detectable in MLN of some *Stat1* ^*-/-*^ mice on 8 dpi (Fig 6A and 6B). RRV titers in the MLN of infected *Rag2* ^*-/-*^ were mainly unchanged from 1 dpi to the conclusion of the experiment on 8 dpi (Fig 6A and 6B), emphasizing the importance of adaptive immunity for virus clearance.

### Both type I and type III IFNs contribute to the restriction of RRV replication in liver

Murine and simian RV strains have also been shown to spread to the liver and replicate in the epithelial lining of the biliary tree [4, 5, 25]. To investigate involvement of type I and type III IFNs in limiting systemic simian RV infection in the liver, we determined hepatic virus titers in RRV-infected single and double IFN receptor-deficient mice. WT and single IFN receptor-deficient mice showed similar levels of virus replication in the liver on 1 dpi (Fig 6C and 6D). RRV titers had declined in WT controls by 3 dpi, but remained significantly elevated in infected *Ifnar1* ^*-/-*^, *Ifnlr1* ^*-/-*^ and *Ifnar1* ^*-/-*^ *Ifnlr1* ^*-/-*^ animals (Fig 6C and 6D). Although virus was cleared from the liver of WT and single IFN receptor-deficient mice by 5 dpi, RRV persisted in the liver of double IFN receptor-deficient *Ifnar1* ^*-/-*^ *Ifnlr1* ^*-/-*^ suckling mice through 8 dpi (Fig 6C and 6D). These data indicate that type I and type III IFNs cooperate to limit RRV spread to and replication in the liver of infected mice. Furthermore, although liver virus titers were similar in 129S6/SvEv WT, *Stat1* ^*-/-*^ and *Rag2* ^*-/-*^ mice on 1, 3 and 5 dpi, both *Stat1* ^*-/-*^ and *Rag2* ^*-/-*^ mice had higher liver virus titers than WT controls on 8 dpi (Fig 6C and 6D). Of note, all mouse strains on the 129S6/SvEv background showed higher virus titers in the liver than any mouse strain on the C57BL/6J background (Fig 6C and 6D), indicating that levels of RRV replication in the liver are also affected by strain-specific genetic factors.

### Antiviral responses against RV in small intestine are mediated by either type I or type III IFNs

Recent studies concluded that intestinal antiviral responses are primarily mediated by type III, rather than type I, IFNs during infection with the homologous murine EDIM-RV [22]. In contrast, we observed that murine EW-RV replication was rather insensitive to the antiviral actions of both type I and type III IFNs in the homologous murine host (Fig 2). On the other hand, the replication of the heterologous simian RRV in suckling mice was substantially restricted by both IFN types (Figs 4–6). In addition, when either IFN was administered systemically, it was able to efficiently stimulate STAT1 activation in IECs of suckling mice (Fig 5A and 5B) and induce antiviral protection against RRV in IFN-pretreated suckling mice (Fig 5E). These findings provided a clear phenotype and biologically relevant RV strain to decipher the relative roles of these two types of IFNs in intestinal innate antiviral responses.

**Fig 7:**
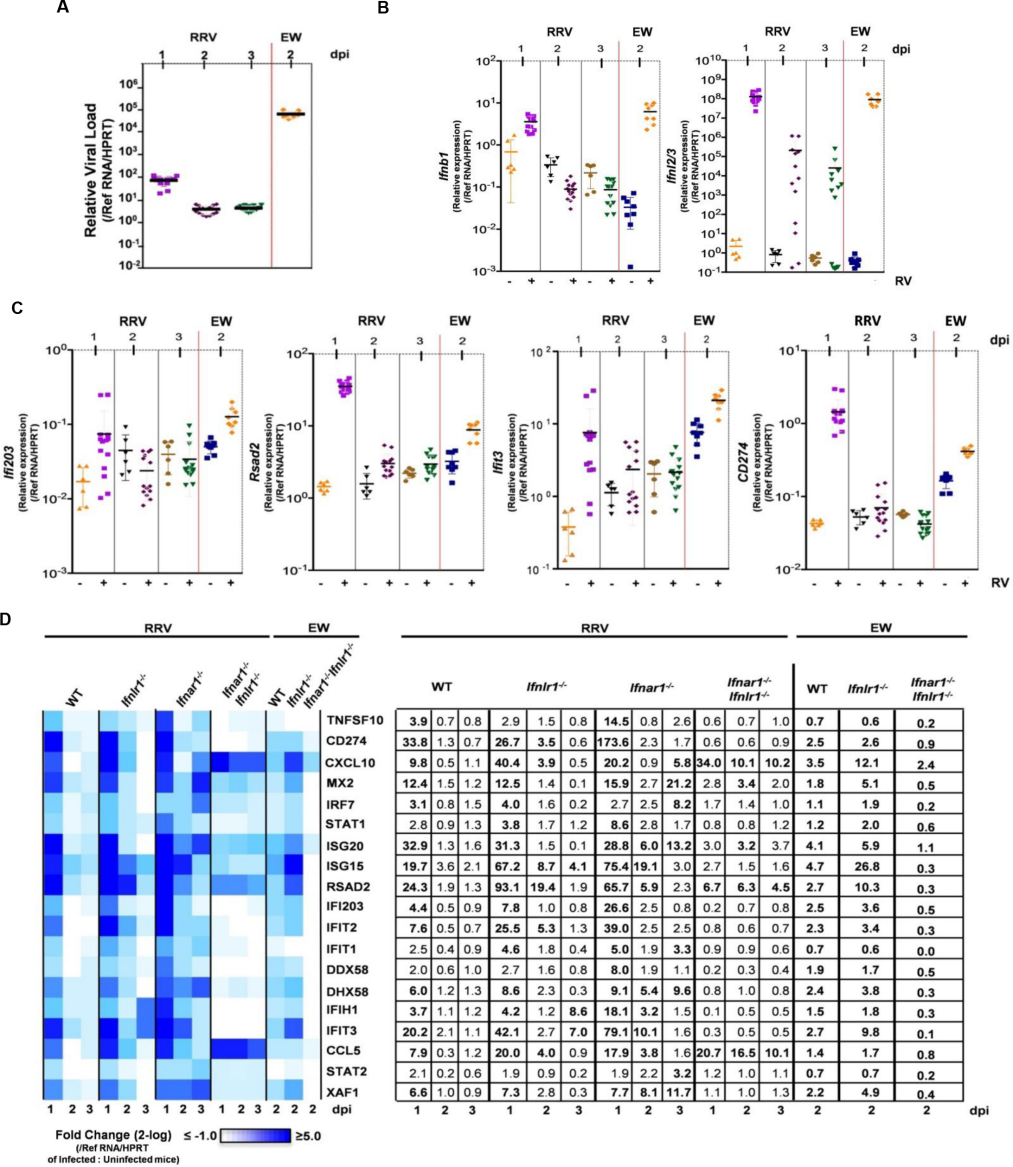
Type I and type III IFNs act redundantly during murine EW-RV and simian RRV infection to establish antiviral programs in small intestine. (A-C) Graph of quantitative RT-PCR detection of (A) RV levels, (B) IFN expression and (C) expression of indicated ISGs in small intestine of RRV-infected WT mice on 1, 2 and 3 dpi and EW-RV-infected WT mice on 2 dpi. Symbols (A-C) duplicate measures from individual mice. (D) Graph of heat map of gene expression in intestinal samples presented as mean fold change in expression in infected mice relative to that of uninfected mice and shown on a 2-log scale. Transcriptional profiling was performed on small intestine samples from RRV and EW-RV-infected WT and single or double IFN receptor-deficient mice. Numbers in the table represent the mean fold change in gene expression on 1, 2 and 3 dpi for RRV, or on 2 dpi for EW-RV-infected, compared to uninfected litters. Changes above 3.0-fold are shown in bold. (n = 12–24 mice per group for RRV and 4–8 mice per group for EW-RV). Replication data in (A) is reformatted from Figs 2 and 4 for comparative purposes. Symbols (A-D) duplicate measures from individual mice. (n = 12–24 mice per group for RRV and 4–8 mice per group for EW-RV).

To perform transcriptional analysis, small intestines of RRV-infected WT mice, as well as pups lacking receptors for either type I or type III IFNs, or both receptors, were isolated 1, 2 and 3 dpi and used for microfluidic qRT-PCR analysis of selected antiviral response transcripts at the bulk whole intestinal level (Fig 7). In these experiments, we also included uninfected animals as well as murine EW-RV-infected WT, *Ifnlr1* ^*-/-*^ and *Ifnar1* ^*-/-*^ *Ifnlr1* ^*-/-*^ mice harvested at a single time point (2 dpi). Of note, we had previously shown that at 16 hpi, despite their substantially different replication capacity *in vivo*, both EW-RV and RRV infections result in comparable levels of ISG and type I IFN induction in bulk intestinal tissues of WT suckling mice [6]. Intestinal EW-RV replication was 1,000- to 10,000-fold greater than that of RRV in WT suckling mice (Fig 7A). Similar to earlier observations at 16 hpi [6], the overall transcriptional levels of EW-RV-induced antiviral cytokines such as IFN-λ and IFN-β, and several IFN-induced antiviral genes such as ISG15 and IFIT3, were similar to, or greater than those induced by RRV on 2 dpi (Figs 7B–7D). We found that infection with the IFN-sensitive RRV strain resulted in the robust induction of several well-defined ISGs, including those encoding IFIT1/2/3, ISG15, ISG20, RSAD2, and Mx2 (Fig 7C and 7D). Notably, transcription of such ISGs was also induced in the absence of either type I or type III IFN receptor in agreement with the ability of both IFN types to trigger STAT1 phosphorylation in IECs, but was almost completely abolished in *Ifnar1* ^*-/-*^ *Ifnlr1* ^*-/-*^ animals (Fig 7D). Thus, type I and type III IFNs drive a set of highly similar antiviral intestinal responses to both EW-RV and RRV, but effectively restrict the replication of only heterologous simian RRV. In the absence of type I and type III IFN signaling, the attenuated RRV-induced transcription of certain ISGs such as *ISG20* and *RSAD2* can be driven by interferon regulatory factors (IRFs) directly or mediated by other virus-induced mechanisms [36, 37]. The absence of types I and type III IFN receptors led to prolonged induction of *CXCL10* and *CCL5* chemokine genes, correlating with extended and increased viral replication. Of interest, genes encoding the anti-microbial proteins REG3B and REG3G (S6 Fig) were induced independently of IFNs by both EW-RV and RRV, with more consistent and higher levels of up-regulation in EW-RV-infected mice. Expression of these genes can be controlled by IL-22, which was recently implicated in host anti-RV restriction [23, 38]. Collectively, transcriptional analysis of bulk intestinal tissues revealed a surprising level of redundancy in the induction of intestinal antiviral responses in suckling mice by type I and type III IFNs.

## Discussion

Type I and type III IFNs are important mediators of innate antiviral defenses. Although these IFNs signal through distinct receptor complexes, the signaling cascades, sets of ISGs up-regulated and biological activities induced in response to these cytokines are almost indistinguishable [8, 9, 13]. For this reason, the relative contributions of type I and type III IFNs to overall antiviral protection of an entire organism can only be investigated with the use of animals deficient in individual and combined specific IFN receptors. Due to the cell-type specific pattern of type III IFN receptor expression that largely limits action of IFN-λs to epithelial cells [19], the target organs for type III IFNs are restricted, whereas type I IFN receptors are expressed ubiquitously, and therefore, expected to evoke antiviral defenses in all tissues and cell types. Nevertheless, there have been several reports demonstrating that mice deficient in STAT1, a transcriptional factor that is critical for signaling of all IFNs, are more susceptible to certain viruses than type I IFN receptor-deficient mice [39, 40]. For example, influenza virus replicated to much higher titers in STAT1 or STAT2 KO mice than in type I IFN receptor-deficient animals [39] suggesting that type III IFNs may also play a role in protecting mice against influenza virus infection. Indeed, it has been demonstrated that protection against influenza A virus replication in airway epithelium can be mediated by either type I or type III IFNs [41–43]. Similarly, it has been shown that RRV replicates better in *Stat1* ^*-/-*^ than in *Ifnar1* ^*-/-*^ suckling mice [6, 25, 26].

It was recently reported that the GI epithelium, and particularly IECs, are protected primarily by type III IFNs in suckling and adult mice, based on the observation that mice deficient in IFN-λ signaling had impaired control of murine RV infection when compared with strain-matched WT and *Ifnar1* ^*-/-*^ mice [22]. In our experiments, on the other hand, the level of murine RV shedding was high and almost indistinguishable in WT or IFN receptor-deficient or STAT1-deficient suckling mice with the exception of slightly delayed but significant clearance differences on 8–9 dpi in *Ifnlr1* ^*-/-*^ and *Stat1* ^*-/-*^ mice; with complete virus clearance from all mouse strains on 10 dpi (Fig 2). In addition, in the current study weight loss and the degree of diarrheal disease were not substantially enhanced in *Ifnlr1* ^*-/-*^ and/or *Stat1* ^*-/-*^ mice when compared to WT suckling mice. These data are inconsistent with the results of Pott et al. and Hernandez et al., which suggested that pathogenesis and susceptibility to murine RV was highly IFN-λ dependent [22, 23]. The basis of the different findings in the studies (Summarized in Table 1) is not readily apparent. Direct comparison of the two murine RV strains used in the two groups of studies indicates that they are highly related or identical in terms of replication capacity in WT and *Stat1* ^*-/-*^ suckling mice (S3 Fig). In addition, although mice, which were used in studies of Pott et al. and Hernandez et al. [22, 23], have a reconstituted functional *Mx1* gene, whereas mice used in other studies possess a non-functional *Mx1* gene, kinetics and magnitude of EW-RV replication were similar in WT suckling mice deficient or reconstituted with the functional *Mx1* gene (S2A and S2B Fig) and profiles of transcriptional IFN and ISG induction were similar (S2C and S2D Fig). Of note, the *Mx1*-reconstituted mice were generated by breeding the A2G-*Mx1* ^*+/+*^ mice onto the *Mx1* ^*-/-*^ C57BL/6 mice for several generation, however the purity of the genetic background of the resulting B6.A2G-*Mx* ^*+/+*^ strain has not been characterized [44]. Moreover, B6.A2G-Mx^+/-^ males, starting from F2 generation, were selected for backcrossing with C57BL/6 females based on their survival of the infection with lethal dose of influenza virus infection. This breeding strategy, in addition for maintaining the *Mx* ^*+/-*^ genotype in breeders, may also put a selective pressure skewing for genes, other than *Mx1*, which also enhance virus resistance. These B6.A2G-*Mx* ^*+/+*^ mice were later crossed with the *Ifnlr1* ^*-/-*^ C57BL/6J mice [41, 45]. It should also be noticed that mice used in the current studies have only a small alteration within the *Ifnlr1* gene, only exon 3 was deleted (Fig 1 and S1 Fig), whereas mice used in studies of Pott et al. and Hernandez et al. [22, 23], have the entire the *Ifnlr1* gene (~20 kb) removed and replaced with the IRES-LacZ/MC1-Neo reporter gene/selection cassette (~5 kb) [45]. This substantial genomic alteration could potentially affect expression of other neighboring genes, particularly the *Il22ra* gene that is located downstream of the *Ifnlr1* gene and encodes one of the IL-22 receptor chains. IL22RA (IL-22R1) shares epithelial cell specific expression pattern with IFN-λR1 and these two adjacent genes may share co-regulatory elements. Therefore, further studies are required to fully characterize and compare the two currently existing *Ifnlr1* ^*-/-*^ mice. In addition, animal diet, microbiota and persistent infections with murine norovirus or helicobacter, which are often present in pathogen-free animal facilities, have been shown to alter innate intestinal antiviral responses [21, 46–48], and therefore could potentially account for some of the observed differences in this and other studies.

**Table 1:** Overview of experimental results with homologous and heterologous RV infection in various KO mouse strains.

Virus strain	Mouse strain	Phenotype	Reference
**Homologous murine RV infection in suckling mice**			
EDIM (G3 P16)	WT (129 Sv/ev)	equal shedding between 5-day-old WT and Stat1^-/-^ mice	[24]
	Stat1^-/-^ (129 Sv/ev)		
EC	WT (129 Sv/ev)	equal titer between 5-day-old WT, *Ifnar* ^*-/-*^, and *Ifnar* ^*-/-*^ *Ifngr* ^*-/-*^ mice	[5, 25, 52]
	Ifnar^-/-^ (B6x129 H2^b^)		
	*Ifnagr* ^*-/-*^ (129 Sv/ev)		
EW	WT (129 Sv/ev)	equal titer between 5-day-old WT and *Stat1* ^*-/-*^ mice	[6, 26]
	*Stat1* ^*-/-*^ (129 Sv/ev)		
EDIM	WT (B6.A2G-*Mx1*)	increased titers and shedding in 4-15-day-old *Ifnlr1* ^*-/-*^ and *Ifnar1* ^*-/-*^ *Ifnlr1* ^*-/-*^ mice, but not WT and *Ifnar1* ^*-/-*^ mice; treatment with recombinant IFN-λ reduces viral titers	[22]
	*Ifnar1* ^*-/-*^ (B6.A2G-*Mx1*)		
	*Ifnlr1* ^*-/-*^ (B6.A2G-*Mx1*)		
	*Ifnar1* ^*-/-*^ *Ifnlr1* ^*-/-*^ (B6.A2G-*Mx1*)		
EDIM	WT (B6.A2G-*Mx1*)	increased titers and shedding in 7-day-old *Ifnlr1* ^*-/-*^ and *Il22* ^*-/-*^ mice, but not WT mice	[23]
	*Ifnlr1* ^*-/-*^ (B6.A2G-*Mx1*)		
	*Il22* ^*-/-*^ (B6)		
EW	WT (B6)	equal titer and shedding between 8-day-old WT, *Ifnlr1* ^*-/-*^, *Ifnar1* ^*-/-*^, *Ifnar1* ^*-/-*^ *Ifnlr1* ^*-/-*^, and *Stat1* ^*-/-*^ mice; *Rag2* ^*-/-*^ mice develop chronic infection	[This study]
	*Ifnar1* ^*-/-*^ (B6)		
	*Ifnlr1* ^*-/-*^ (B6)		
	*Ifnar1* ^*-/-*^ *Ifnlr1* ^*-/-*^ (B6)		
	WT (129 Sv/ev)		
	*Stat1* ^*-/-*^ (129 Sv/ev)		
	*Rag2* ^*-/-*^ (129 Sv/ev)		
**Heterologous simian RV infection in suckling mice**			
RRV	WT (129 Sv/ev)	increased titers in 5-day-old *Stat1* ^*-/-*^ mice	[5, 6, 25, 26]
	*Stat1* ^*-/-*^ (129 Sv/ev)		
RRV	WT (B6)	increased titers and shedding in 8-day-old *Ifnlr1* ^*-/-*^ and *Ifnar1* ^*-/-*^ mice; synergistically increased titers and shedding in *Ifnar1* ^*-/-*^ *Ifnlr1* ^*-/-*^ and *Stat1* ^*-/-*^ mice; *Rag2* ^*-/-*^ mice develop chronic infection	[This study]
	*Ifnar1* ^*-/-*^ (B6)		
	*Ifnlr1* ^*-/-*^ (B6)		
	*Ifnar1* ^*-/-*^ *Ifnlr1* ^*-/-*^ (B6)		
	WT (129 Sv/ev)		
	*Stat1* ^*-/-*^ (129 Sv/ev)		
	*Rag2* ^*-/-*^ (129 Sv/ev)		

Of note, we did observe declining responsiveness of IECs to type I IFNs in adult mice (Fig 5C and 5D), but saw robust IFN signaling in suckling mice IECs following treatment with either IFN-α or IFN-λ (Fig 5A and 5B). We also observed that low constitutive levels of STAT activation are present in IECs of WT adult mice, but not in *Ifnlr1* ^*-/-*^ mice (Fig 5D), suggesting that IFN-λ signaling seems to be maintained in IECs and may contribute to the well documented decreased ability of murine EDIM-RV to replicate as efficiently in adult as in suckling mice [49]. These findings are interesting but unlikely to fully account for the differences observed between the two sets of studies (Table 1) as both of these were carried out in suckling mice. Recent studies on intestinal antiviral immunity have shown compartmentalized effects of type I and type III IFNs, where LPCs were protected only by type I IFNs, whereas type III IFNs were indispensable for restricting reovirus or RV replication in IECs [20, 22]. Using a direct immunohistochemical assay of IFN-triggered STAT1 activation in IECs and LPCs, we also observed that LPCs responded only to type I IFNs by STAT1 activation (Fig 5). However, we observed that administration of either type I or type III IFNs could induce the restriction of RRV replication in the small intestine of suckling mice with one caveat: the responsiveness of IECs to type I IFNs was substantially more pronounced in neonatal mice, where RV disease is present, than in adults (Fig 5), where RV replication is restricted and RV associated disease is absent [50, 51].

RV infections are remarkably host specific. Homologous RVs replicate to significantly higher levels in the intestines of homologous hosts, require much lower doses to cause disease and spread more efficiently among non-immune susceptibles than heterologous RVs. As with several other virus infections, it has been shown that RV host-range restriction is, in large part, determined by the different efficiency of homologous versus heterologous RVs in antagonizing the host IFN response [6, 25, 26]. Indeed, we also observed that murine EW-RV replicated in mice much more efficiently than heterologous RRV (Fig 7A). However, the higher EW-RV load induced similar magnitude of IFN (Figs 2E, 4E and 7B) and ISG (Figs 2F, 4F, 7C and 7D) responses as the much lower load of RRV. Similar findings were reported when responses to EW-RV and RRV were compared at 16 hpi in suckling mouse intestines, and reinforce the notion that homologous murine RVs have evolved highly effective measures to circumvent host innate immune responses in order to replicate efficiently and cause diarrheal disease that promotes virus dissemination [6, 25, 26]. The robust murine RV replication was not appreciably augmented in IFN receptor or STAT1 deficient suckling mice (Fig 2 and [6, 24–27]). Therefore, in order to study the relevant importance of type I and type III IFNs in initiating and propagating intestinal innate immune responses, we used heterologous simian RRV that has been previously shown to be much more sensitive than homologous murine RV to innate antiviral defenses in suckling mice [6, 25, 26]. RRV replicates but only poorly in the WT suckling mouse intestine and is unable to spread from inoculated to susceptible litter mates while murine EW-RV is more virulent, replicates to much higher levels in the mouse intestine, and spreads very efficiently among litter mates.

Our results revealed that RRV replicated much more efficiently in either type I or type III IFN receptor-deficient suckling mice, and the complete lack of IFN responses in *Ifnar1* ^*-/-*^ *Ifnlr1* ^*-/-*^ or *Stat1* ^*-/-*^ mice allowed RRV replication to proceed to even higher titers with delayed clearance in comparison to single IFN receptor-deficient mice (Fig 4). Accordingly, pretreatment of suckling mice with either IFN-α or IFN-λ 6 h prior to RRV infection suppressed intestinal RRV replication to the similar extent and combined IFN-α or IFN-λ pretreatment provided a similar level of protection as pretreatment with either type of IFN alone (Fig 5E). On the other hand, pretreatment of suckling mice with type I or II interferon had no effect on homologous murine RV replication or diarrheal disease [52]. In addition, transcriptional analysis in the whole intestine of RV-infected suckling mice revealed that classical ISGs were induced to similar levels in either type I or type III IFN receptor-deficient animals (Fig 7), confirming independent and overlapping actions of type I and type III IFNs in the intestinal antiviral defense of suckling mice.

RRV was also able to replicate more efficiently in MLN of *Ifnar1* ^*-/-*^ or *Ifnar1* ^*-/-*^ *Ifnlr1* ^*-/-*^ mice than in WT or *Ifnlr1* ^*-/-*^ mice (Fig 6A and 6B), suggesting that at this site, type I and not type III IFNs were primarily responsible for controlling RV replication. Both single and double IFN receptor KO mice demonstrated impaired control of RRV replication in the liver (Fig 6C and 6D), but only *Ifnar1* ^*-/-*^ *Ifnlr1* ^*-/-*^ mice failed to clear RRV from the liver by 5 dpi (Fig 6C and 6D). Of note, RRV replication was better controlled by type III IFNs at earliest times post infection, because increased viral transcription was detected only on 1 dpi, and was quickly suppressed by 2 dpi in *Ifnar1* ^*-/-*^ mice, whereas viral transcripts were still elevated on 2 and 3 dpi in *Ifnlr1* ^*-/-*^ mice (Fig 4D), suggesting a somewhat more prominent role of type III IFNs in controlling intestinal RV replication and this correlated with more efficient *Ifnl2/3* induction by RV than those of type I IFNs (Figs 4E and 7B, and S4 Fig). More prolonged intestinal RRV replication in *Ifnlr1* ^*-/-*^ mice might give virus more time to disseminate to and replicate in other organs. Nevertheless, we observed distinct patterns of RRV spread and replication in MLNs and liver, the former controlled primarily by type I IFNs (Fig 6A and 6B) and the latter by both IFN types (Fig 6C and 6D).

Collectively, these data demonstrate that neither IFN alone or together play a significant role in regulating the robust replication and disease phenotypes of the homologous murine RV in suckling mice. On the other hand, these studies clearly demonstrate that both type I and type III IFNs are required for optimal antiviral protection of the GI tract of suckling mice against the heterologous simian RRV infection, and that both IFN types independently contribute to innate antiviral defenses within the intestinal mucosal compartment (Figs 4, 5 and 7) and cooperate to restrict extra-intestinal RRV replication in other tissues (Fig 6). Our studies also identified a reduced sensitivity of IECs but not LPCs to the effects of type I but not type III IFNs as mice mature. Overall, our findings highlight a multi-faceted complexity of the virus-host interactions and reveal a well-orchestrated spatial and temporal tuning of innate antiviral responses in the intestinal tract where two types of IFNs through distinct patterns of their expression and distinct but overlapping sets of target cells coordinately regulate antiviral defenses. Our findings also highlight the fact that the antiviral capacity of the various IFNs can vary very significantly between strains of the same virus in a host dependent manner.

## Materials and Methods

### Mice

Conventional specific pathogen-free (SPF) WT C57BL/6J mice were purchased from Jackson Laboratory. Mice lacking functional IFN-λ receptor (*Ifnlr1* ^*-/-*^) were generated in the laboratory. Recombineering techniques were used to create a KO targeting vector that contained exon 3 of the mouse *IFNLR1* gene flanked with two LoxP sites and ~10 kb arms for homologous recombination (Fig 1A). A neo (G418-selection) cassette flanked with the FRT sites was introduced in front of the LoxP site in intron 4. The accuracy of all modified sequences within the targeting vector was verified by sequencing. Bruce4 mouse embryonic stem (ES) cells from C57BL/6J strain were transfected with the targeting vector, and G418-resistant ES clones were selected and screened by Southern blotting for correct integration of the targeting fragment (S1A and S1B Fig). Chromosomal DNA was obtained from G418-resistant ES clones, digested with *Eco*RV restriction endonuclease, subjected to Southern blotting with a hybridization probe corresponding to exons 1 and 2 of the mouse *IFNLR1* gene that are positioned outside of the left arm for homologous recombination (S1A Fig). Twenty three clones were selected and their DNA was digested with *AflIII* restriction endonuclease, and Southern blotting was performed with a probe corresponding to exons 5, 6 and 7 that are outside of the right arm for homologous recombination (S1B Fig). One of the clones with the correct integration pattern at both 3' and 5’ ends was used for the generation of chimeric mice, and subsequently mice homozygous for the integration cassette. First, the neo cassette was eliminated by crossing the chimeric mice with C57BL/6J mice transgenic for the CMV promoter-driven flipase (Jackson Laboratory, Stock # 009086). Mice homozygous for the deletion of the neo cassette were selected, followed by the selection against the flipase gene. These mice were then crossed with C57BL/6J mice transgenic for the CMV promoter-driven Cre recombinase (Jackson Laboratory, Stock # 006054), and mice homozygous for the deletion of the IFN-λR1 exon 3 and lacking the *Cre* gene were selected. These IFN-λ receptor-deficient animals were crossed with C57BL/6J mice lacking functional type I IFN receptor (*Ifnar1* ^*-/-*^ mice) in the laboratory of Jörg Fritz at McGill University; and *Ifnar1* ^*-/-*^ and *Ifnar1* ^*-/-*^ *Ifnlr1* ^*-/-*^ mice were provided for these studies. Congenic B6.A2G*-Mx1* mice carrying intact *Mx1* alleles [44] and EDIM-RV isolate were provided by P. Staeheli. All mouse strains on C57BL/6J background were maintained at SPF barrier facility at NJMS, Rutgers. Mouse strains on 129S6/SvEv background were described previously [25] and maintained in the vivarium at the Veterinary Medical Unit of the Palo Alto VA Health Care System.

### RV infection

Eight-day-old suckling mice were orally inoculated with 10^4^ DD_50_ of the murine EW-RV strain or 4x10^6^ FFU of the simian RRV strain. The EW-RV strain was derived following serial suckling mouse passage from the original E. Kraft EDIM-RV isolate [33]. From 2 to 12 dpi for EW-RV infection or from 2 to 8 dpi for RRV infection, animals were examined daily for the occurrence of diarrheal disease. The percentage of diarrhea among inoculated littermates during the course of infection for each group was recorded. To measure the effects of RV infection and IFN deficiency on suckling mouse body weight gain, EW-RV or RRV-infected or non-infected WT 129S6/SvEv, *Stat1* ^*-/-*^ and *Rag2* ^*-/-*^ mice were weighed daily during the course of experiments. Daily mouse weight ratio was calculated for each infected mouse as weight of infected mouse (g) / mean weight of uninfected control mice (g) of the same age. Fecal specimens (approximately 10–20 μl) were collected from EW-RV-infected suckling mice into pre-weighed eppendorf tubes. Samples were stored at -80ºC prior to fecal EW-RV shedding detection by ELISA. At indicated day post EW-RV or RRV infection, a number of mice from each experimental group were sacrificed for tissue collection and histology.

### IFN treatments

All IFNs were injected intradermally in adult or suckling mice. Human hybrid IFN-αA/D and mouse IFN-λ2 were used at the concentrations indicated in the figure legends. Human IFN-αA/D was previously shown to be highly active on many mouse cell types *in vitro* and *in vivo* [53].

### Polarized cultures of IECs

Human SW-1116 cells (ATCC CCL-233) were plated at confluency onto transwell filters and cultured for 56 days (media was changed every other day) until epithelial layer of well-polarized epithelial cells with high trans-epithelial resistance (TER > or = 2000 ohm/cm2) was established. In parallel, SW-1116 cells were also grown in continuously proliferating cultures on regular plates. The cells were left untreated or treated at the apical or basolateral surfaces with various amounts of IFN-α or IFN-λ as indicated. At 72 h, the cells were collected, and levels of MHC class I antigen expression were evaluated by flow cytometry.

### Virus titration by focus forming unit assay

At various time points post RRV infection, suckling mice were anesthetized, and tissue samples from liver, MLN, and small intestine were collected and stored at -80°C. Before assay, the thawed tissue samples were individually weighed and made to 10% (wt/vol) suspensions with serum free M199. Samples were homogenized in 5 ml polypropylene tubes and the homogenates were activated with trypsin (10 μg/ml) for 1 h at 37°C in a 5% CO_2_ incubator. Total homogenates were centrifuged at 1,500 rpm for 10 min and the supernatants were serially diluted in serum free M199. MA-104.1 cells (ATCC CRL-2378.1) were inoculated in a 24-well plate with 0.1 ml of diluted supernatant. After absorption for 1 h at 37°C in a 5% CO_2_ incubator, cells were re-fed with 500 μl 10% FBS M199 supplemented with 2 mM L-glutamine and penicillin/streptomycin (100 μg/ml / 100 I.U.) and cultured for 24 h. The cells were then fixed with 10% phosphate-buffered formalin for 30 min. Viral antigenic focus detection was accomplished by incubation with rabbit anti-rotaviral hyperimmune serum for 1 h, then alkaline phosphatase (AP)-conjugated goat anti rabbit IgG (Invitrogen) for 1 h, then the AP substrate BCIP/NBT (5-bromo-4-chloro-3-indolyl phosphate/nitro blue tetrazolium) (Sigma). Between each step, wells were washed twice with PBS-TA (PBS containing with 0.1% Triton X-100, 0.1% BSA, 0.1% sodium azide). The positive cells were enumerated and virus titers were expressed as focus forming unit (FFU) per gram of tissue.

### Detection of EW-RV shedding in feces by ELISA

Fecal EW-RV antigen shedding was measured as previously described [54]. Briefly, fecal samples were made to 10% wt/vol suspensions with PBS. Ninety-six-well polystyrene high binding plates (E&K Scientific) were coated with guinea pig anti-rotavirus hyperimmune serum. After washing and blocking with 5% BLOTTO (wt/vol fat free power milk in PBS) suspended stool samples were added to the plates for overnight incubation at 4°C. The plates were washed and rabbit anti-rotavirus hyperimmune serum was added to the plates. The plates were washed, and horseradish peroxidase (HRP)-conjugated goat anti-rabbit immunoglobulin G (IgG) antibody (γ chain specific, Thermo scientific) was added to the plates. TMB (3,3’,5,5’-Tetramethylbenzidine) substrate (Kirkegaard & Perry Laboratories) was used for the color reaction. A serial dilution of a standard RRV stock was used in each plate to control the level of color development. The absorption at A450 nm was measured with an ELISA reader (Bio-Tek Instruments). The fecal viral antigen shedding data were expressed as optical density (OD) values.

### Immunohistochemistry

The small intestines were formalin-fixed and paraffin-embedded. Antigen retrieval was performed on deparaffinized 5 micron sections which were then incubated for 5 min with Super Block (ScyTek #AAA999), and 10 min in 3% H_2_O_2_ to block the endogenous peroxidase activity. Sections were then incubated at 4°C overnight with polyclonal goat anti-rotavirus antiserum (NCDV; Meridian, LS; 1:500) or monoclonal rabbit anti-phospho-STAT1 (Tyr701; 58D6) (Cell Signaling; 1:500). Slides were washed 2 times in PBST (0.05% Tween-20 in PBS), then incubated at room temperature for 30 min with UltraTek anti-Goat biotinylated antibody (Ready to Use) (ScyTek #AGL125) or UltraTek anti-rabbit biotinylated antibody (Ready to Use) (ScyTek #ABK125), followed by a 20 min room temperature incubation with UltraTek Streptavidin/HRP (Ready to Use). NovaRED substrate solution (Vector, SK-4000) was used as a substrate. After immunostaining, tissue sections were washed twice in water and counterstained with Mayer’s haematoxylin and Scott’s bluing buffer.

### RT-PCR analysis and immunoblotting

Mice were sacrificed and sections of the small intestines (all tissues (bulk) of the small intestine) were collected and lysed in Trizol (Life Technolgies) on ice. Total RNA was extracted following the manufacturer’s instructions and subjected to DNAse digestion before use in qRT-PCR. Synthesis of cDNA and subsequent microfluidics PCR on the Fluidigm platform was done as described earlier [6]. Serial 10-fold dilutions of mouse reference RNA (Agilent) were run in duplicate for each PCR run. Relative gene expression in infected and uninfected mouse intestinal samples was derived using the 2dCt method [6] with reference RNA serving as a calibrator and HPRT as housekeeping control.

Cell lysates were collected in lysis buffer containing protease and phosphatase inhibitors. Equal amounts of total protein was separated on 7.5% SDS-PAGE gels, transferred to Nitrocellulose 0.45 μm membrane (BIO-RAD), and subsequently probed with antibody against phosphorylated STAT1 (pY701; BD #612133) and β-actin (Sigma #A5441).

### IFN ELISA and bioassay

The supernatants of intestinal homogenates of RRV-infected mice were prepared as described above and assayed for IFN-λ protein using commercially available ELISA (R&D Systems), and for IFN-α/β protein by bioassay as previously described [55].

### Statistics

Sigmaplot 12.5 or GraphPad Prism software was used for data analysis. Virus levels in tissue were determined by either focus forming unit assay or real time quantitative RT-PCR, and was analyzed with one-way ANOVA and Bonferroni multiple comparison test with the log-transformed viral titers.

### Ethics statement

All animal studies were approved by the NJMS Institutional Animal Care and Use Committee (Protocol 13009C0316) and the VA Palo Alto Health Care System Institutional Animal Care and Use Committee (Protocol GRH0022/GRH1397) and carried out in accordance with the recommendations in the Guide for the Care and Use of Laboratory Animals of the National Institutes of Health.

## Supporting Information

S1 Fig Screening of ES cell clones containing the floxed exon 3 of the *Ifnlr1* gene. Southern blot analyses were performed to selected ES cell clones with the correct integration of the targeting vector. (A) Genomic DNA from one hundred clones was digested with *EcoRV* restriction endonuclease and subjected to Southern blotting with a probe corresponding to exons 1 and 2 of the *Ifnlr1* gene; positive clones that are numbered demonstrate two closely positioned bands hybridizing with the probe. (B) Twenty three clones with the correct integration of the left arm were selected and their DNA was digested with *AflIII* restriction endonuclease, and Southern blotting was performed with a probe corresponding to exons 5, 6 and 7. Clones with correct integration at the 3' end demonstrate two bands hybridizing with the probe. Four ES clones that were selected for the generation of chimeric mice are marked with double +. (C) Kidney cells were obtained from 5-day-old WT or *Ifnlr1* ^*-/-*^ pups. The cells were left untreated (closed histograms) or treated for 48 h with IFN-α (green histograms) or IFN-λ (red histograms) and IFN-mediated induction of MHC class I molecules was evaluated by flow cytometry.
(TIF)

S2 Fig The presence of the *Mx1* gene does not affect insensitivity of EW-RV to the action of IFNs. Eight-day-old suckling conventional *Mx1*-deficient C57BL/6J mice (n = 8 mice) and *Mx1*-reconstituted B6.A2G-*Mx1* mice (n = 8 mice) were orally infected with 10^4^ DD_50_ EW-RV. (A) Stool samples were collected daily from 2 to 12 dpi, EW-RV shedding in stool samples was determined by ELISA and expressed as OD unit, and kinetics of fecal EW-RV shedding were drawn. (B-D) Quantitative RT-PCR detection of (B) EW-RV levels, (C) IFN expression and (D) expression of ISGs in small intestine of EW-RV-infected mice on 2 dpi. Each symbol (B-D) represents an individual mouse; horizontal lines indicate the mean (± SEM).
(TIF)

S3 Fig EDIM-RV and EW-RV strains demonstrate similar fecal shedding. (A, B) Eight-day-old WT and STAT1 KO suckling mice on 129S6/SvEv background (A) and WT C57BL/6J suckling mice (B) were orally inoculated with 10^4^ DD_50_ of indicated viral strains produced from intestinal homogenates from pooled infected suckling mouse intestines. Fecal samples were collected on 2, 4 and 6 dpi and assayed by ELISA. OD values > 0.1 are positive.
(TIF)

S4 Fig RRV infection triggers higher levels of type III IFNs than type I IFNs in suckling mice. Eight-day-old suckling WT, *Ifnar1* ^*-/-*^, *Ifnlr1* ^*-/-*^ and *Ifnar1* ^*-/-*^ *Ifnlr1* ^*-/-*^ suckling mice on C57BL/6J background were orally infected with 4x10^6^ FFU RRV. Small intestines were collected on 1 dpi, tissue homogenates were prepared and used for IFN-λ ELISA.
(TIF)

S5 Fig The lack of *Ifna* gene responses after RRV infection. (A-C) Quantitative RT-PCR detection of IFN-α1 (A), IFN-α4 (B), and IFN-α5 (C) expression in small intestine of RRV-infected WT and various IFN receptor-deficient mice on 1, 2 and 3 dpi. Symbols duplicate measures from individual mice. (n = 12–24 mice per group for RRV and 4–8 mice per group for EW-RV). Horizontal lines indicate the mean (± SEM).
(TIF)

S6 Fig Genes encoding the anti-microbial proteins REG3B and REG3G are induced by RV independently of IFNs. (A-D) Quantitative RT-PCR detection of REG3B (A and C) and REG3C expression (B and D) in small intestine on 2 dpi of EW-RV (A and B) or on 1, 2 and 3 dpi of RRV (C and D) infected WT and various IFN receptor-deficient mice. Symbols duplicate measures from individual mice. (n = 12–24 mice per group for RRV and 4–8 mice per group for EW-RV). Horizontal lines indicate the mean (± SEM).
(TIF)
