# Correction: Distinct Roles of Type I and Type III Interferons in Intestinal Immunity to Homologous and Heterologous Rotavirus Infections

**DOI:** 10.1371/journal.ppat.1005726

**Published:** 2016-06-15

**Authors:** Jian-Da Lin, Ningguo Feng, Adrish Sen, Murugabaskar Balan, Hsiang-Chi Tseng, Constance McElrath, Sergey V. Smirnov, Jianya Peng, Linda L. Yasukawa, Russell K. Durbin, Joan E. Durbin, Harry B. Greenberg, Sergei V. Kotenko

The email address given for the last author is incorrect. The correct email address for Sergei Kotenko is: kotenkse@njms.rutgers.edu
