# Supplementary figures and images for: Distinct Roles of Type I and Type III Interferons in Intestinal Immunity to Homologous and Heterologous Rotavirus Infections
Source: PLoS Pathog. 2016 Apr 29;12(4):e1005600. doi: 10.1371/journal.ppat.1005600 (PMC4851417; doi:10.1371/journal.ppat.1005600)

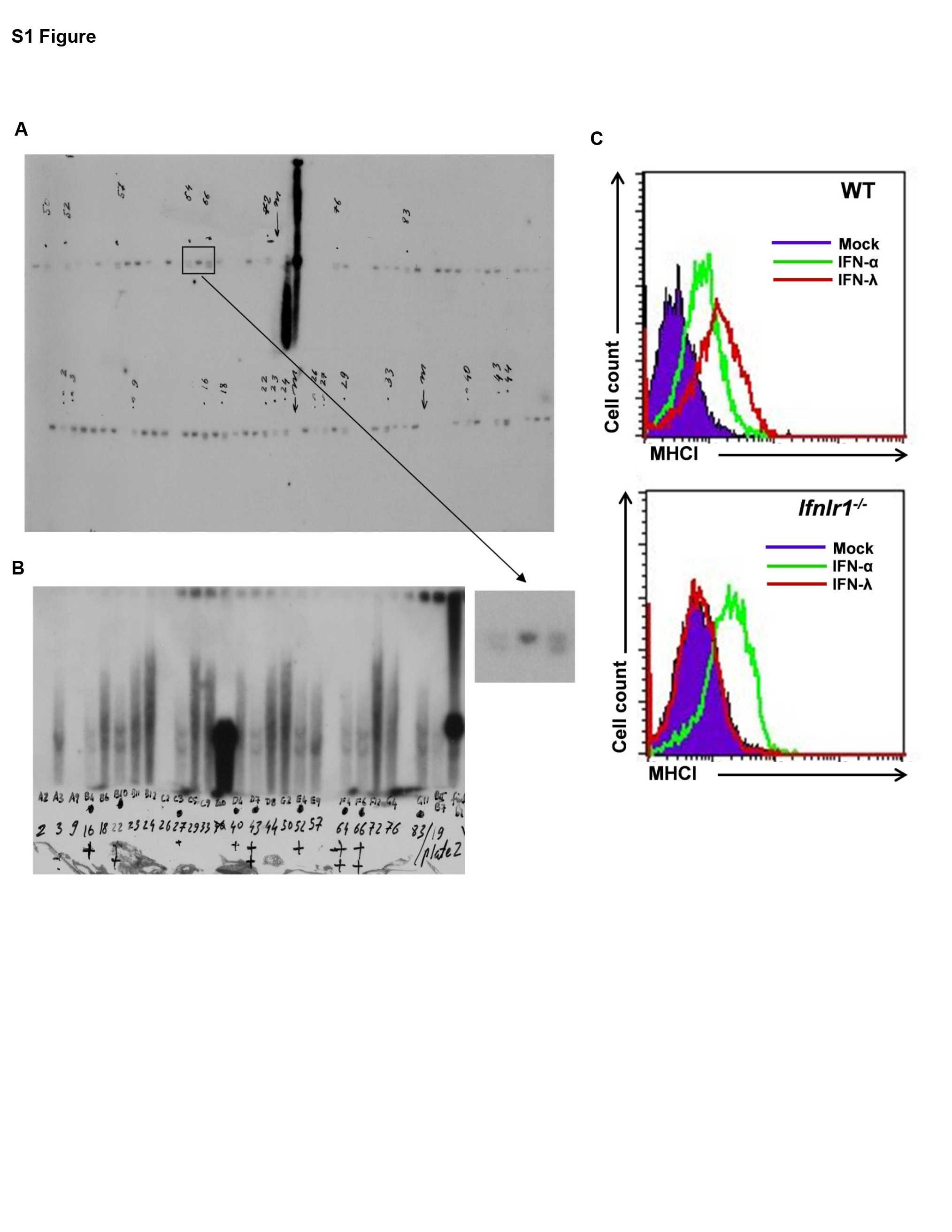

Supplement: S1 Fig — Southern blot analyses were performed to selected ES cell clones with the correct integration of the targeting vector. (A) Genomic DNA from one hundred clones was digested with EcoRV restriction endonuclease and subjected to Southern blotting with a probe corresponding to exons 1 and 2 of the Ifnlr1 gene; positive clones that are numbered demonstrate two closely positioned bands hybridizing with the probe. (B) Twenty three clones with the correct integration of the left arm were selected and their DNA was digested with AflIII restriction endonuclease, and Southern blotting was performed with a probe corresponding to exons 5, 6 and 7. Clones with correct integration at the 3' end demonstrate two bands hybridizing with the probe. Four ES clones that were selected for the generation of chimeric mice are marked with double +. (C) Kidney cells were obtained from 5-day-old WT or Ifnlr1 -/- pups. The cells were left untreated (closed histograms) or treated for 48 h with IFN-α (green histograms) or IFN-λ (red histograms) and IFN-mediated induction of MHC class I molecules was evaluated by flow cytometry. (TIF) [file ppat.1005600.s001.tif]

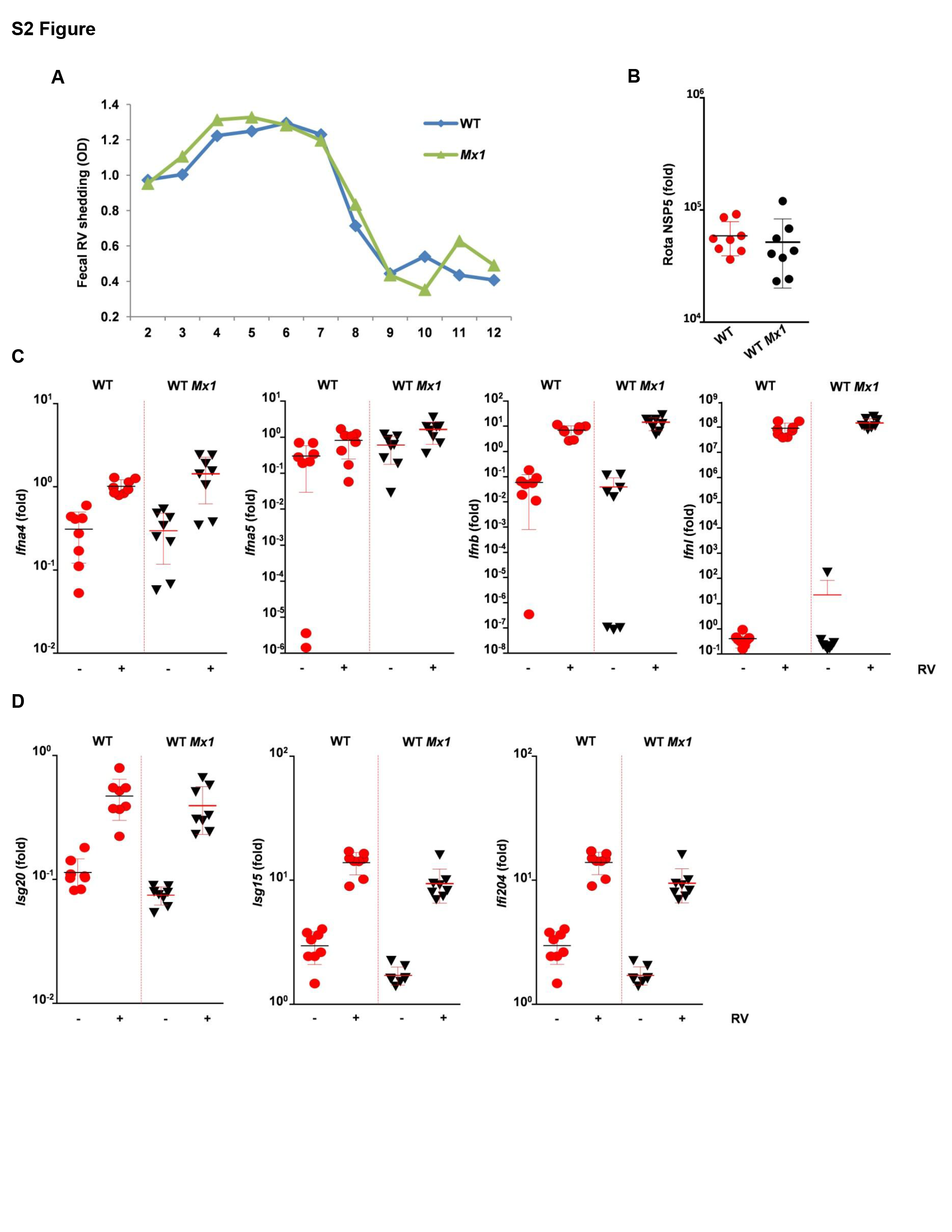

Supplement: S2 Fig — Eight-day-old suckling conventional Mx1-deficient C57BL/6J mice (n = 8 mice) and Mx1-reconstituted B6.A2G-Mx1 mice (n = 8 mice) were orally infected with 104 DD50 EW-RV. (A) Stool samples were collected daily from 2 to 12 dpi, EW-RV shedding in stool samples was determined by ELISA and expressed as OD unit, and kinetics of fecal EW-RV shedding were drawn. (B-D) Quantitative RT-PCR detection of (B) EW-RV levels, (C) IFN expression and (D) expression of ISGs in small intestine of EW-RV-infected mice on 2 dpi. Each symbol (B-D) represents an individual mouse; horizontal lines indicate the mean (± SEM). (TIF) [file ppat.1005600.s002.tif]

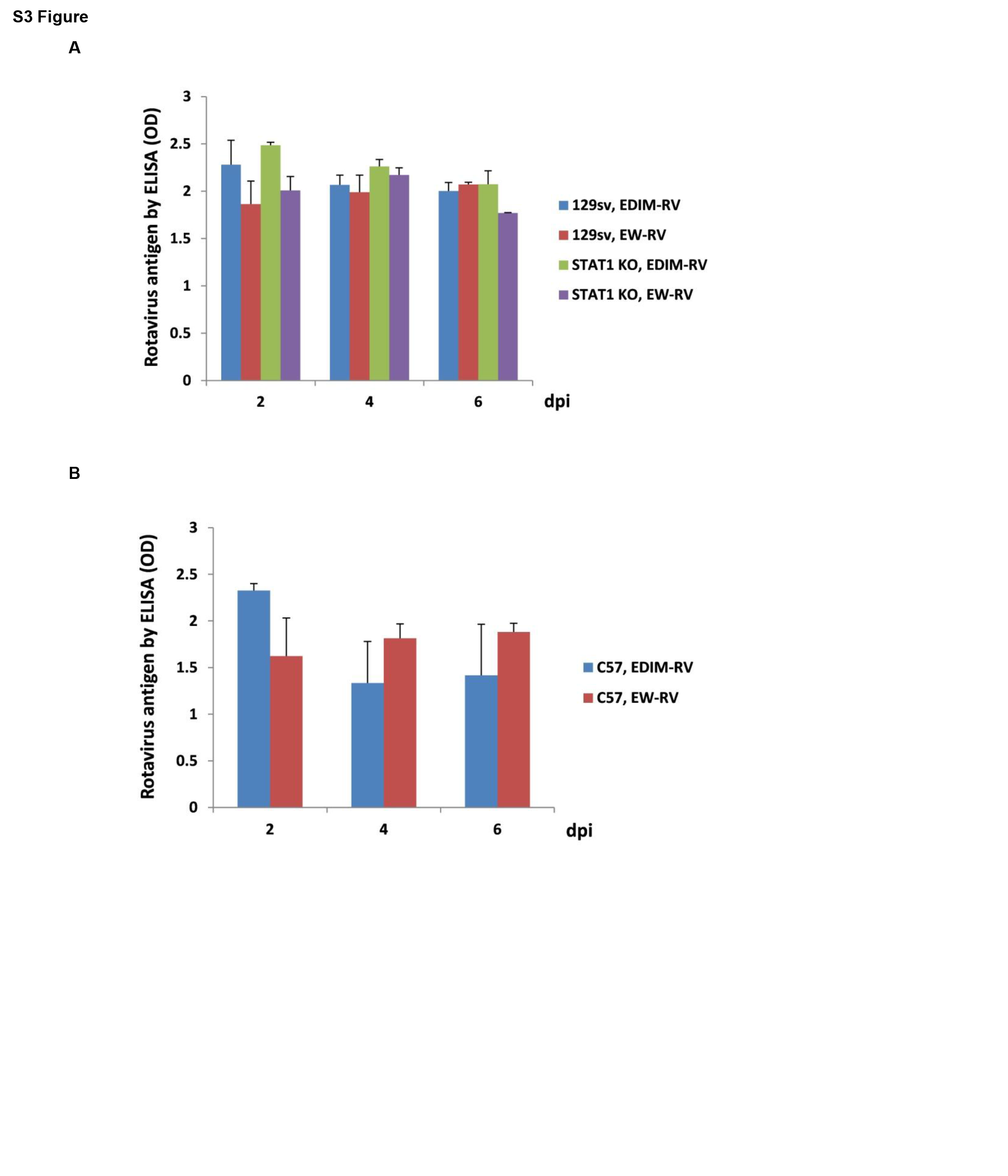

Supplement: S3 Fig — (A, B) Eight-day-old WT and STAT1 KO suckling mice on 129S6/SvEv background (A) and WT C57BL/6J suckling mice (B) were orally inoculated with 104 DD50 of indicated viral strains produced from intestinal homogenates from pooled infected suckling mouse intestines. Fecal samples were collected on 2, 4 and 6 dpi and assayed by ELISA. OD values > 0.1 are positive. (TIF) [file ppat.1005600.s003.tif]

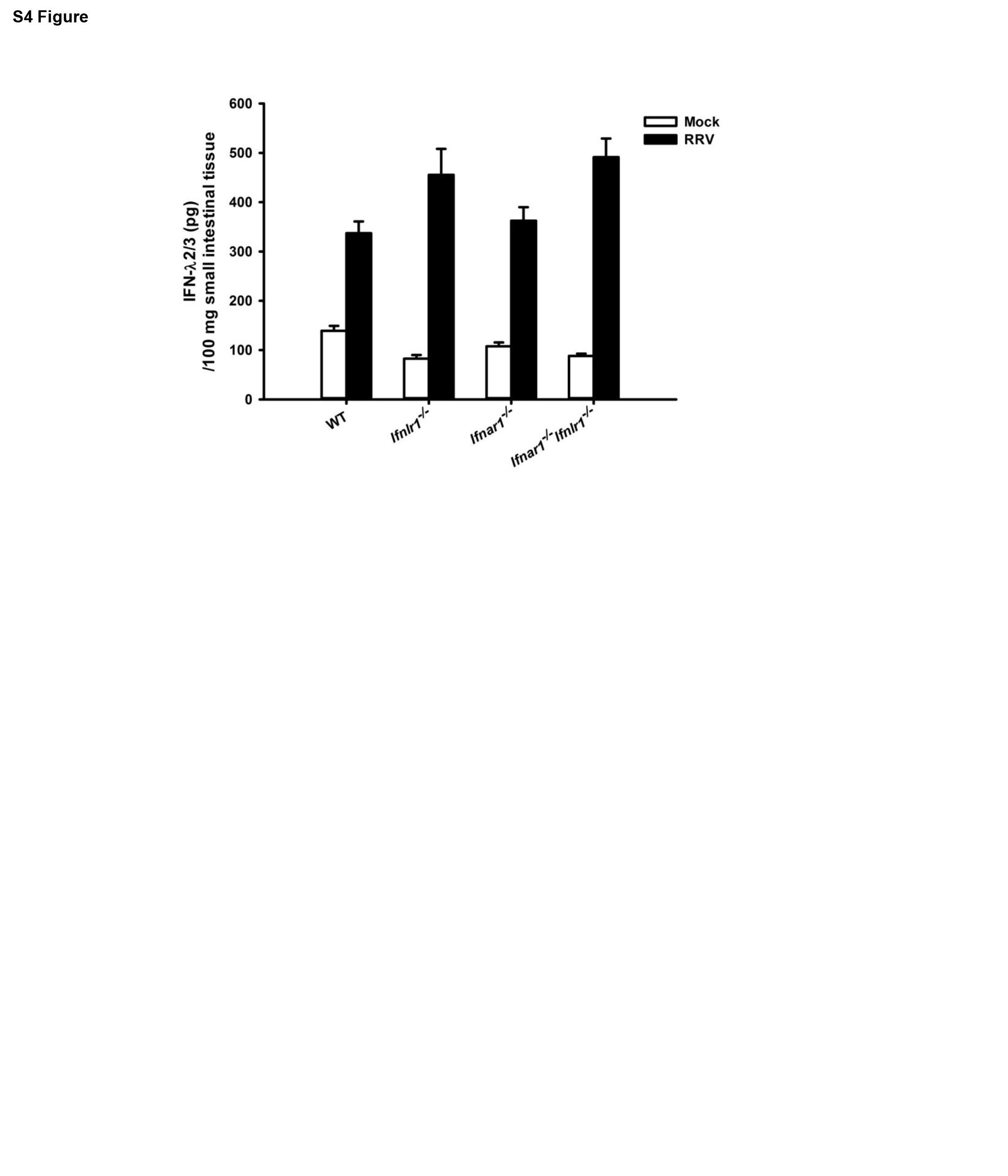

Supplement: S4 Fig — Eight-day-old suckling WT, Ifnar1 -/-, Ifnlr1 -/- and Ifnar1 -/- Ifnlr1 -/- suckling mice on C57BL/6J background were orally infected with 4x10^6^ FFU RRV. Small intestines were collected on 1 dpi, tissue homogenates were prepared and used for IFN-λ ELISA. (TIF) [file ppat.1005600.s004.tif]

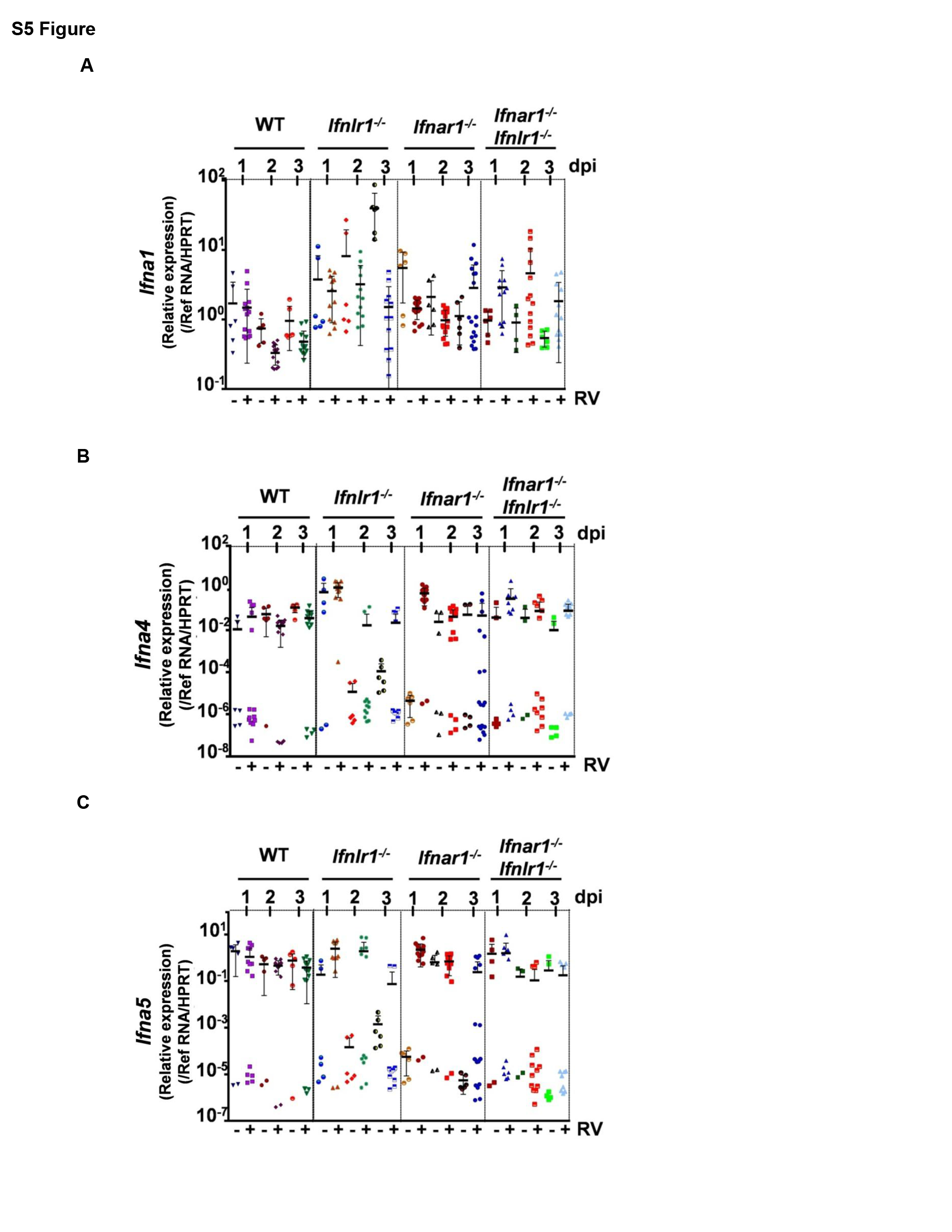

Supplement: S5 Fig — (A-C) Quantitative RT-PCR detection of IFN-α1 (A), IFN-α4 (B), and IFN-α5 (C) expression in small intestine of RRV-infected WT and various IFN receptor-deficient mice on 1, 2 and 3 dpi. Symbols duplicate measures from individual mice. (n = 12–24 mice per group for RRV and 4–8 mice per group for EW-RV). Horizontal lines indicate the mean (± SEM). (TIF) [file ppat.1005600.s005.tif]

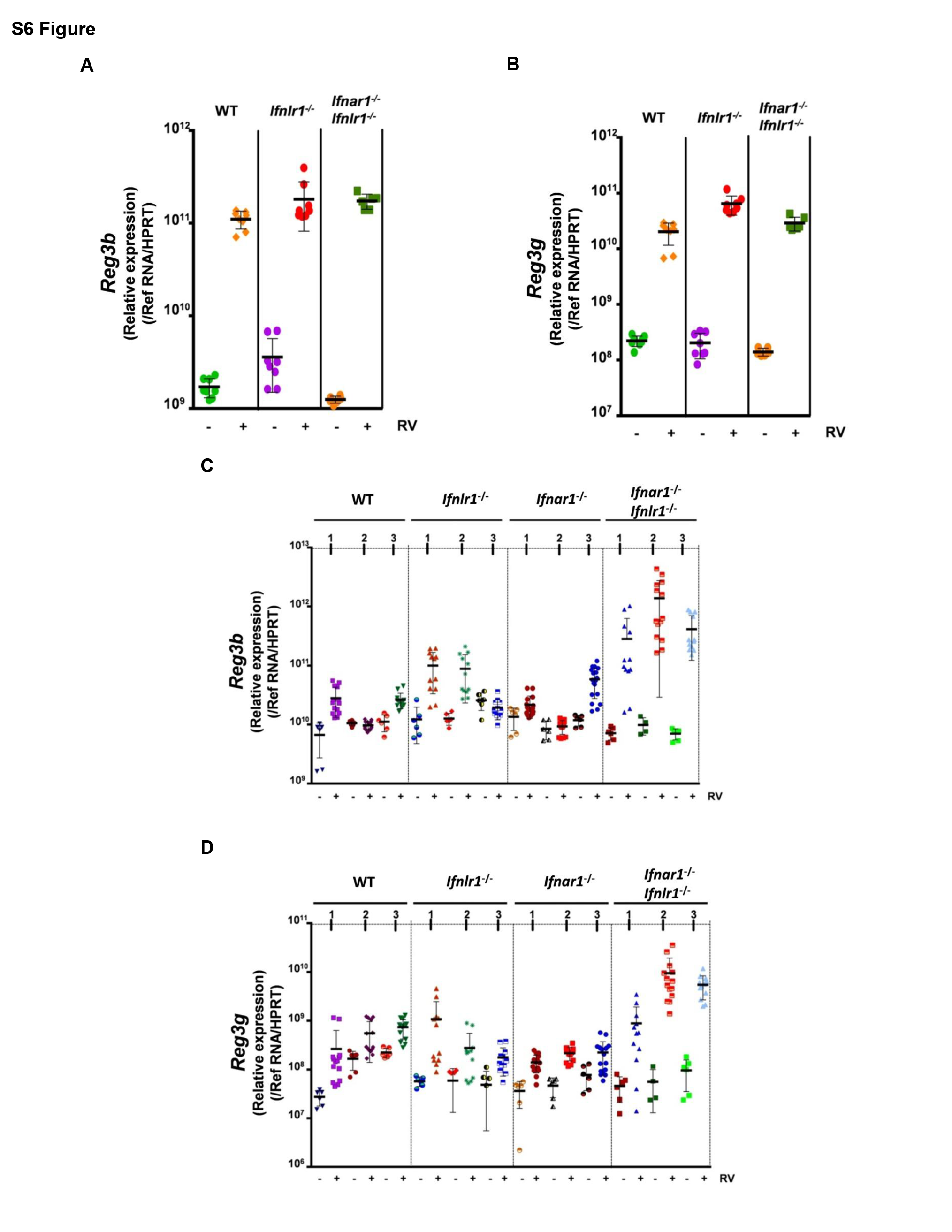

Supplement: S6 Fig — (A-D) Quantitative RT-PCR detection of REG3B (A and C) and REG3C expression (B and D) in small intestine on 2 dpi of EW-RV (A and B) or on 1, 2 and 3 dpi of RRV (C and D) infected WT and various IFN receptor-deficient mice. Symbols duplicate measures from individual mice. (n = 12–24 mice per group for RRV and 4–8 mice per group for EW-RV). Horizontal lines indicate the mean (± SEM). (TIF) [file ppat.1005600.s006.tif]
